# In silico studies evidenced the role of structurally diverse plant secondary metabolites in reducing SARS-CoV-2 pathogenesis

**DOI:** 10.1038/s41598-020-77602-0

**Published:** 2020-11-25

**Authors:** Hariprasad Puttaswamy, Hittanahallikoppal Gajendramurthy Gowtham, Monu Dinesh Ojha, Ajay Yadav, Gourav Choudhir, Vasantharaja Raguraman, Bhani Kongkham, Koushalya Selvaraju, Shazia Shareef, Priyanka Gehlot, Faiz Ahamed, Leena Chauhan

**Affiliations:** Centre for Rural Development and Technology, Indian Institute of Technology Delhi, New Delhi, Delhi 110016 India

**Keywords:** Computational biology and bioinformatics, Drug discovery

## Abstract

Plants are endowed with a large pool of structurally diverse small molecules known as secondary metabolites. The present study aims to virtually screen these plant secondary metabolites (PSM) for their possible anti-SARS-CoV-2 properties targeting four proteins/ enzymes which govern viral pathogenesis. Results of molecular docking with 4,704 ligands against four target proteins, and data analysis revealed a unique pattern of structurally similar PSM interacting with the target proteins. Among the top-ranked PSM which recorded lower binding energy (BE), > 50% were triterpenoids which interacted strongly with viral spike protein—receptor binding domain, > 32% molecules which showed better interaction with the active site of human transmembrane serine protease were belongs to flavonoids and their glycosides, > 16% of flavonol glycosides and > 16% anthocyanidins recorded lower BE against active site of viral main protease and > 13% flavonol glycoside strongly interacted with active site of viral RNA-dependent RNA polymerase. The primary concern about these PSM is their bioavailability. However, several PSM recorded higher bioavailability score and found fulfilling most of the drug-likeness characters as per Lipinski's rule (Coagulin K, Kamalachalcone C, Ginkgetin, Isoginkgetin, 3,3′-Biplumbagin, Chrysophanein, Aromoline, etc.). Natural occurrence, bio-transformation, bioavailability of selected PSM and their interaction with the target site of selected proteins were discussed in detail. Present study provides a platform for researchers to explore the possible use of selected PSM to prevent/ cure the COVID-19 by subjecting them for thorough in vitro and in vivo evaluation for the capabilities to interfering with the process of viral host cell recognition, entry and replication.

## Introduction

Coronaviruses (CoV) are spherical or pleomorphic, enveloped, non-segmented particles contain positive-sense single-stranded RNA^1^. There are several types of low to high pathogenic CoV which cause mild to severe respiratory symptoms. In general, it is classified under four genera such as α, β, γ, and δ CoV. α and β-CoV are reported to cause fatal respiratory tract infections in mammals, among which Severe Acute Respiratory Syndrome-CoV (SARS-CoV) is grouped under β-CoV. Whereas, γ, and δ CoV are reported to infect birds^2^. In last three decades, several CoV virus related diseases have been frequently reported in human and animals such as human CoV OC43, 299E, Bovin CoV, Canine CoV, Feline CoV, Porcine CoV, etc., indicating their increasing competence to expand their host range^3^. But, their capabilities to cause fatality in human was realized with the SARS outbreak occurred during 2002 and 2003^4,5^. Recent addition to this list is, Coronavirus disease 19 (COVID-19) caused by SARS-CoV-2 which is much more fatal to humans than their earlier versions.

COVID-19 was first spotted in a seafood market of Wuhan city, Hubei Province of China, and now it had reached all continents across the globe^6^. Based on genomic sequence evidence, bat CoV RaTG13 shares 96.2% similarity with SARS-CoV-2. Hence, bats are suspected as a primary source of SARS-CoV-2 and infected humans through several intermediate hosts^7,8^. However, this hypothesis is yet to be proved. The SARS-CoV-2 virus causes SARS by the onset of pneumonia, acute respiratory distress syndrome (ARDS) and multi-organ dysfunction. Moreover, the SARS-CoV-2 enters the human body through the mouth and other openings and spreads primarily through droplets, saliva, or discharges from the nose/ mouth of an infected person after sneezing or coughing^9^. On 11th March 2020, the World Health Organization (WHO) declared COVID-19 as a pandemic. As on 27th July, 2020 the global COVID-19 data show 1,61,14,449 confirmed cases and 6,46,641 confirmed deaths (http://www.who.int).

At present, there are no pharmaceutical products approved by the Food and Drug Administration (FDA) as safe and effective for the treatment of COVID-19. Further, as precautionary measures, surveillance borders, personal hygiene, social distancing, lockdown, supportive care and personal protective equipment are practiced across the world to prevent disease spreading among the communities. Preliminary in vitro studies and clinical trials carried out by scientists on COVID-19 patients disclosed the effectiveness of hydroxychloroquine, an anti-malarial drug in combination with azithromycin, a broad spectrum anti-bacterial drug in reducing the disease severity^10^. Similarly, the use of several off-label medicines (such as lopinavir-ritonavir, favipiravir, remdesivir, ribavirin, anti-Interleukin-6 inhibitors, etc.) have been suggested to treat COVID-19 as potential investigational drugs. But, on 4th July, 2020, based on the results obtained from the solidarity trials, WHO ruled out the possible use of hydroxychloroquine and lopinavir-ritonavir as anti-COVID-19 drug (WHO, 2020). Similarly, researchers across the world attempting to identify/ develop drug molecule targeting several viral pathogenicity factors such as, spike protein-human Angiotensin-converting enzyme 2 (hACE2) mediated viral entry, main protease (M^pro^), papain-like protease 2 (PLP2), RNA-dependent RNA polymerase (RdRp), SARS-CoV helicase, etc^11^. Any new drug molecule requires thorough scientific evaluation in terms of their anti-viral potencies, efficacy, bioavailability, adverse effect on non-target site, safety, different stages of preclinical and clinical trials, etc. before available for the public use.

Plants are known to inherently contain a large number of structurally diverse secondary metabolites which are developed during the course of evolution, primarily as defence mechanisms against herbivores and predators, mediating pollination and for protection against abiotic stress^12^. The variability of metabolites can be seen across different plant families, genera, species and different parts of the same plant species^13^. The concentrations of PSM vary according to the growth stages and in response to biotic and abiotic stress to which plant exposed. The development of drugs from phytopharmaceuticals is a trending approach to look for eco-friendly therapeutic molecules with no or minimal side-effects. This time-bound situation requires an efficient and effective method to develop therapeutics which disables the virus molecular machinery. Considering the safety of the users, any conventional drug discovery plan is a time-consuming process that sometimes takes decades to complete. Thus, repurposing the already available FDA approved drugs, use of plant-based herbal medicines, or edible plant parts rich in anti-viral PSM are other strategies appears to be promising under current situations.

In silico or computational approaches are algorithm-based virtual screening methods developed for screening a large number of molecules in shorter time and identification of probable potent drug candidate. In recent research, 1,903 approved drugs were virtually screened through molecular docking, and binding free energy calculations suggested nelfinavir as a potential inhibitor against SARS-CoV-2^14^. Similarly, phytomolecules such as kaempferol, quercetin, luteolin-7-glucoside, demethoxycurcumin, naringenin, apigenin-7-glucoside, oleuropein, curcumin, catechin, and epicatechin-gallate have been reported as potential viral M^pro^ inhibition^15^. Elfiky^16^ reported Ribavirin, Remdesivir, Sofosbuvir, Galidesivir, and Tenofovir as potent drug candidates against RdRp of SARS-CoV-2 through molecular docking studies.

Most of the earlier PSM based virtual screening performed were either limited by the number of PSM, structural diversity of test ligands and number of target proteins. Hence, the present study is aimed to (i) creating a structurally diverse PSM library, (ii) finding potent PSM which binds to the target site of selected proteins/ enzymes with low binding energy (BE), (iii) studying structural and functional relation of top scored PSM, and (iv) analyzing the physicochemical characters and bioavailability of selected PSM using SwissADME.

## Results and discussion

The PSM library contains 4,704 molecules collected from 203 plant species belong to diverse plant families (Supplementary File 1). Over 22,000 docking reactions were run (which include replications, to confirm the activity of several molecules with BE) using 4,704 ligands against four selected target proteins/ enzymes involved in host cell recognition, entry and replication of SARS-CoV-2. Upon molecular docking, a wide range of BE was obtained for all four target proteins. The obtained results were arranged in ascending order of BE. For the ease of the study, we selected the top 268 molecules against M^pro^ and 250 molecules for all other target proteins for further analysis (Supplementary File 2) viz*.*, structural similarity and activity relationship, and physicochemical characterization to evaluate their drug-likeness. Binding energy, physicochemical characters and bioavailability score (BAS) of ten top ranked molecules against four target proteins are compiled in Table 1.

**Table 1:** Binding energy of top ranked plant secondary metabolites against four targets of SARS-CoV-2 pathogenesis and their physicochemical properties.

Code	Name	BE	MW	RB	HA	HD	TPSA	MLogP	PGP	GI	LV	BAS
**SARS-CoV-2 Spike protein**												
B0162-10327320	Bismahanine	− 9.1	692.8	7	4	4	90.50	6.18	Yes	Low	2	0.17
C0572-100920596	Coagulin N	− 9.1	648.7	4	12	6	192.44	− 0.36	Yes	Low	3	0.17
A0297-16162334	Arecatannin A3	− 8.9	1443.2	9	30	25	551.90	− 3.21	Yes	Low	3	0.17
C0563-15970528	Coagulin K	− 8.9	616.7	4	10	4	151.98	1.16	Yes	Low	1	0.55
G0134-16129878	Tannic acid	− 8.9	1701.2	31	46	25	777.98	− 4.24	Yes	Low	3	0.17
K0070-101721039	Kamalachalcone C	− 8.8	530.5	4	8	4	147.14	1.92	No	Low	1	0.55
A0155-5281600	Amentoflavone	− 8.7	538.4	3	10	6	181.80	0.25	No	Low	2	0.17
P0479-16398499	Pseudojervine	− 8.7	587.7	3	9	5	137.71	1.02	Yes	High	1	0.55
F0096-643975	Flavin adenine dinucleotide	− 8.6	785.5	13	20	9	382.55	− 3.77	Yes	Low	3	0.11
G0075-156783	Graecunin E	− 8.6	1047.1	11	22	12	335.06	− 3.68	Yes	Low	3	0.17
**SARS-CoV-2 RNA-dependent RNA polymerase (RdRp)**												
E0189-3564542	Eriodictyol-7-O-rutinoside	− 9.9	596.5	6	15	9	245.29	− 3.24	Yes	Low	3	0.17
N0007-442431	Narirutin	− 9.7	580.5	6	14	8	225.06	− 2.77	Yes	Low	3	0.17
H0135-191266	Hippomannin A	− 9.6	634.4	6	18	11	318.50	− 2.90	Yes	Low	3	0.17
I0135-5318569	Isoginkgetin	− 9.5	566.5	5	10	14	159.80	0.63	No	Low	1	0.55
K0010-5491813	*Kaempferol	− 9.5	600.4	7	15	9	257.04	− 2.30	No	Low	3	0.17
M0284-44259428	Myricetin 3-rutinoside	− 9.5	626.5	6	17	11	289.66	− 4.35	Yes	Low	3	0.17
R0047-441943	Rotundioside B	− 9.5	1184.3	15	26	13	428.71	− 3.89	Yes	Low	3	0.11
T0047-73179	Tellimagradin I	− 9.5	786.5	9	22	13	385.26	− 3.08	Yes	Low	3	0.17
A0245-5281599	Agathisflavone	− 9.4	538.4	3	10	6	181.80	0.25	No	Low	2	0.17
E0140-119058016	Emblicanin A	− 9.4	782.5	6	22	12	374.26	− 2.33	Yes	Low	3	0.11
**Human transmembrane serine protease (TMPRSS2)**												
G0154-14982	Glycyrrhizic acid	− 9.5	822.9	7	16	8	267.04	0.02	Yes	Low	3	0.11
C0387-366355	cis-Miyabenol C	− 9.4	680.7	6	9	7	160.07	3.43	No	Low	2	0.17
P0126-124025	Proanthocyanidin A2	− 9.2	576.5	2	12	9	209.76	0.14	No	Low	3	0.17
G0038-131752181	Granatin B	− 9.1	952.6	3	27	14	450.25	− 3.45	Yes	Low	3	0.17
H0134-101601938	Hippophaenin B	− 9.1	1104.7	7	31	19	542.17	− 3.90	Yes	Low	3	0.11
C0126-101710863	3-Caffeoyl-5-Feruloylquinic Acid	− 9	530.4	10	12	16	200.28	− 0.15	Yes	Low	3	0.11
B0138-183757	3,3′-Biplumbagin	− 8.9	374.3	1	6	2	108.74	0.62	No	High	0	0.55
A0245-5281599	Agathisflavone	− 8.9	538.4	3	10	6	181.80	0.25	No	Low	2	0.17
A0156-362574	Aromoline	− 8.9	594.7	2	8	2	83.86	3.37	No	High	1	0.55
C0453-6324923	Chrysophanein	− 8.9	416.3	3	9	5	153.75	− 1.26	Yes	Low	0	0.55
**SARS-CoV-2 Main protease (Mpro)**												
H0349-3663	Hypericin	− 10.4	504.4	0	8	6	155.52	1.36	No	Low	2	0.17
A0155-5281600	Amentoflavone	− 9.7	538.4	3	10	6	181.80	0.25	No	Low	2	0.17
T0163-44584734	Terflavin B	− 9.7	784.5	8	22	13	385.24	− 2.83	Yes	Low	3	0.17
M0522-21593828	Mudanpioside J	− 9.6	630.5	11	14	5	199.90	− 0.04	Yes	Low	2	0.17
Q0019-44259101	Quercetin 3,5-digalactoside	− 9.6	626.5	7	17	11	289.66	− 4.62	No	Low	3	0.17
V0041-168165	Vescalagin	− 9.6	934.6	0	26	16	455.18	− 3.23	Yes	Low	3	0.17
G0227-5271805	Ginkgetin	− 9.5	566.5	5	10	4	159.80	0.63	No	Low	1	0.55
I0135-5318569	Isoginkgetin	− 9.5	566.5	5	10	14	159.80	0.63	No	Low	1	0.55
C0163-44256718	Cyanidin 3,5-diglucoside	− 9.4	611.5	7	16	11	272.59	− 3.82	No	Low	3	0.17
D0307-15922818	*Delphinidin	− 9.4	611.5	8	14	9	239.97	− 1.18	No	Low	3	0.17

Only top 10 ranked molecules against each target are represented here. Details of all the plants secondary metabolites studied are available in Supplementary Files 2 and 3.

BE, binding energy (Kcal/mol); MW, molecular weight (g/mol); RB, number of rotatable bonds; HA, number of H-bond acceptors; HD, Number of H-bond donors; TPSA, total polar surface area (Å^2^); MLogP, predicted octanol/water partition coefficient; PGP-S, pgp substrate; GI, GI tract crossing, LV, Number of Lipinski’s rule violation; BAS: bioavailability score.

*Kaempferol 3-O-(6′'-galloyl)-beta-D-glucopyranoside; *Delphinidin-3-O-(6-p-coumaroyl) glucoside.

### SARS-CoV-2 spike protein

Spike protein is a class I fusion protein present within the envelope as a homotrimer and consists of three S1-S2 heterodimers. The receptor-binding domain (RBD) is located on the head of S1^17^ and binds with the cellular receptor hACE2^18^. Any PSM interacts with these selected Amino acid residues (AARs) of spike protein by forming multiple H bonds and other interactions with lower BE may interfere with the spike protein -hACE2 interactions, thereby preventing the early recognition of host cell by SARS-CoV-2.

**Figure 1:**
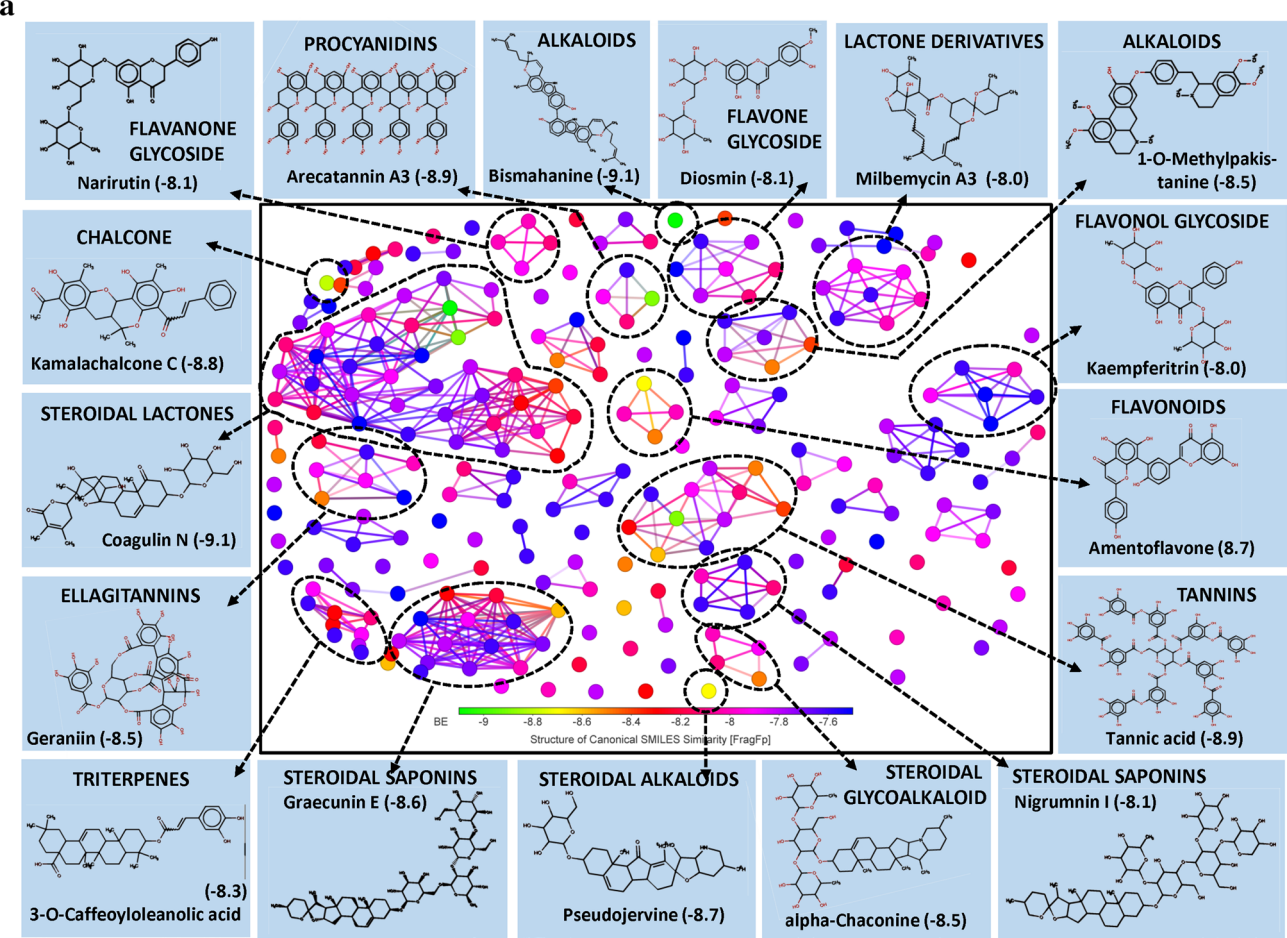

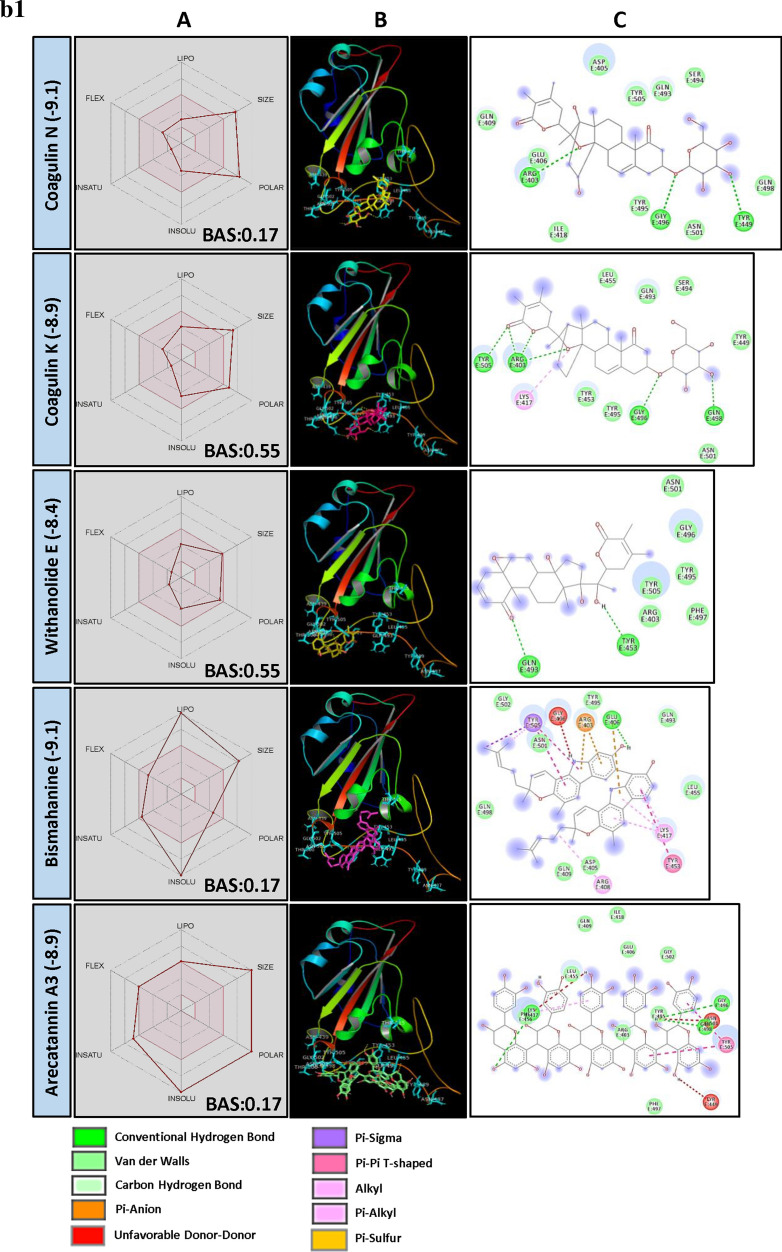

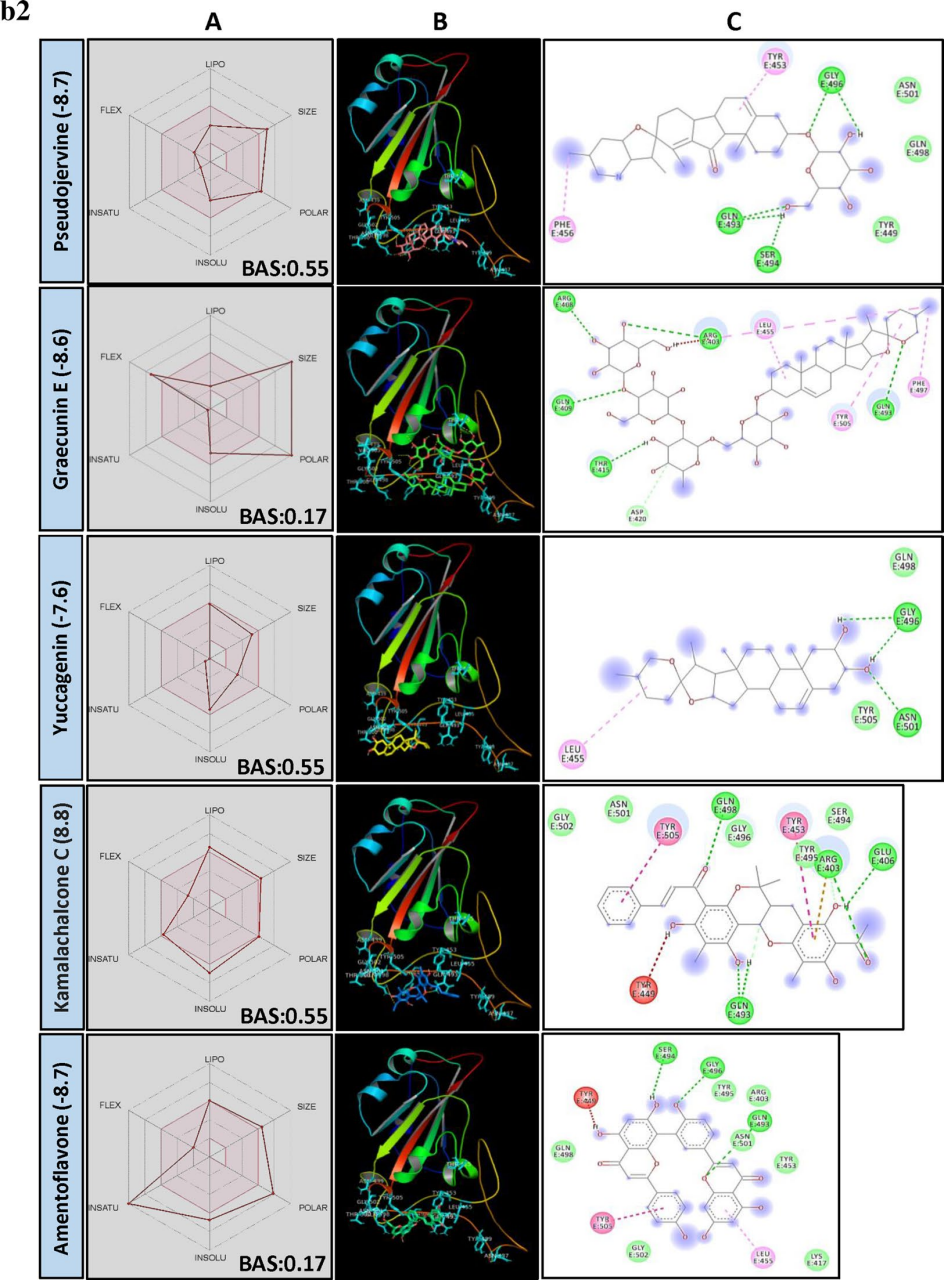
(**a**) Structural activity relationship: correlation of canonical SMILES structure similarity (data points are joined by colored lines) and binding energy (represented in different color shades of data point) of selected plant secondary metabolites (PSM) evaluated against SARS-CoV-2 spike protein using Data Warrior software. Structurally similar molecules are grouped in dotted lines and a representative molecule with low binding energy (kcal/mol) (values in parenthesis) is represented in box. More than 50% of the PSM among top 250 molecules studied belong to triterpenoids and their derivatives, with > 14% Sterol lactones. (**b**) Data analysis of selected PSM against SARS-CoV-2 spike protein. (**A**) Bioavailability radar chart representing lipophilicity (LIPO), Molecular weight (SIZE), Topological polar surface area (POLAR), Solubility (INSOLU), Flexibility (FLEX) and Saturation (INSATU) along with Bioavailability score (BAS) of selected molecules, (**B**) 3D visualization of protein–ligand interaction using PyMOL (selected amino acid residue of target site of protein are colored in cyan, and (**C**) 2D visualization of different types of interactions between ligand and target site of protein using Discovery Studio software (different types of interactions are represented in color codes).

Interestingly, the large pool of PSM found interacting with the exposed surface of spike protein was found belonging to class triterpenoids. More than 50% of the PSM among top 250 molecules belong to triterpenoids and their derivatives, largely include triterpenes and sterols. Here, sterol lactones alone represent > 14% of total PSM (Fig. 1a). Few biflavonoids, flavonoid glycoside and hydrolysable tannins were also showed promising results.

Coagulins from *Withania coagulans* (Stocks) Dunal were recorded lower BE with target AARs of the spike protein. Similar observations were made with structurally similar triterpene and steroid, i.e., steroidal lactones, steroidal saponins, steroidal glycoalkaloids, triterpene glycosides, triterpene saponins, and triterpene sterols. Coagulin N recorded a least of − 9.1 BE followed by Coagulin K (BE -8.9). Coagulin N forms H bond with ARG403, TYR449 and GLY496 of spike protein, whereas Coagulin K forms H bond with ARG403, GLY496, GLN498 and TYR505, which may interfere with viral host cell recognition process. Also, both Coagulin N and Coagulin K recorded 0.55 BAS, and passed all the Lipinski's rule of drug-likeness except molecular weight (MW) (Fig. 1b, Supplementary File 2).

Withanolides are naturally occurring C_28_-Steroidal lactone triterpenoids build on an intact or rearranged estrogen framework^19–21^, commonly found in *Withania somnifera* (L.) Dunal and *W. coagulans* (Stocks) Dunal. Withanolide E recorded the least BE of − 8.4 followed by Withanone (-8.3), Ashwagandhanolide (-8.3) and Withastramonolide (− 8.2) (Supplementary File 2). Withanolide E showed H bond formation with GLN493 and TYR453 AARs of spike protein (Fig. 1b, Supplementary File 2) with BAS 0.55. Withanolides are smaller in molecular weight with high GI absorption and follow all the characters of the drug-like molecules as per Lipinski's rule (Supplementary File 3).

Saponins are naturally occurring plant glycosides found in a wide range of plant species. They are high molecular weight amphiphilic compounds having triterpenoids and steroid aglycon as lipophilic moiety and sugars as hydrophilic moiety^22^. Another class of basic steroidal saponins contains nitrogen analogues of steroid sapogenins as aglycones. The bio-transformation of saponins mainly occurs at intestine with the aid of gut microbes leading to the generation of the rare low molecular weight saponins containing no or lower number of sugar moiety^23^. These hydrolyzed products are higher in bioavailability and bioactivity compared to their parental compounds^24–26^. In our study, Graecunin E (from *Trigonella foenum-graecum* L.)^27^ recorded a least of − 8.6 BE and found interacting with THR415 and GLN493 AARs of spike protein (Fig. 1b, Supplementary File 2). Other Graecunin related compounds, Trigofoenoside E1 (BE − 8.2), Uttronin B (BE -8.3), Stigmasteryl glucoside (BE − 7.9) and Yuccagenin (BE − 7.6) also showed promising results. It was noticed that BE of above-mentioned molecules was related to their number of the sugar moiety. As the number of sugar moiety reduces, BE also found reducing. However, with the loss of sugar moiety, their bioavailability was increasing as observed between Greacunin E and Yuccagenin (aglycon form) (Fig. 1b, Supplementary Files 2 and 3). Also, Yuccagenin was found forming H bonds with GLY496 and ASN501 AARs of spike protein which is crucial to interact with AARs of hACE2 and found fulfilling all drug-likeness characters as per Lipinski's rule (Fig. 1b).

Bismahanine, a carbazole alkaloid isolated from leaves of *Murraya koenigii* (L.) Spreng^28,29^ and some other Rutaceae members^30^. Bismahanin and related compounds are reported for their broad biological activities such as anti-oxidant, anti-diabetic, anti-inflammatory, anti-microbial, anti-cancerous, anti-viral, etc.^31–33^. In our studies, physicochemical properties of Bismahanin revealed it as a low polar, non-soluble, high MLOGP value and high molecular weight PSM according to Lipinski's rule of drug-likeness, which reduces its bioavailability (BAS 0.17). The molecule interacted with residue GLU406 through H bonds and showed Van der Waals interaction with other residues (TYR495, LEU455, ASN501 and GLY502) of spike protein which are not reported to involve in interaction with AARs of hACE2 (Fig. 1b, Supplementary File 2). Pseudojervine, a steroidal alkaloid was first isolated from the rhizome of *Veratrum album* L. by Wright and Luff^34^ which is regularly used in Chinese traditional medicine. In the present study, Pseudojervin recorded − 8.7 BE and found interacting with AARs GLN493, GLY496 and SER494 of spike protein through H bonds. Also, it passed all the Lipinski’s rule except molecular weight (587.74 g/mol) and recorded 0.55 BAS (Fig. 1b, Supplementary Files 2 and 3).

Tannic acid is a high molecular weight polyphenolic compound, highly soluble in water (low lipophilicity). Its GI absorption is almost nil in its original form. However, it is hypothesized that bio-transformed products may enter into plasma and exert biological activities which still need a thorough study. In our study, Tannic acid recorded lower BE of − 8.9, and some of the structurally related hydrolysable tannins such as Strictinin (BE − 8.6), Punicalagin (BE − 8.5), Terchebulin (BE − 8.4), Tercatain (BE − 8.3), Terflavin A (BE − 8.1) also showed promising results (Supplementary File 2).

Arecatannin A3 (a condensed tannins) contains epicatechin-epicatechin-epicatechin-epicatechin-catechin as their basic structure and in its original form it is poorly bioavailable because of their unfavourable physicochemical properties (Supplementary File 3). But, its bio-transformed products, especially monomers, dimers or trimers, may show a varied degree of bioavailability and bioactivity. Their monomers catechin and epicatechin are reported as bioavailable better than their parental molecules^35^. However, their dimer and trimers are poorly bio-available^36^. In our studies, none of the catechin or epicatechin, and their derivatives were appeared in top 250 PSM rank indicating their inability to bind an open surface of the spike protein. Some Arecatannin related molecules such as Arecatannin B1 (BE − 8.1), Proanthocyanidine A-6 (BE − 7.9) and Proanthocyanidine A1 (BE − 7.6) also found interacting with spike protein (Supplementary File 2).

Kamalachalcone C is a dimeric chalcone first isolated from *Mallotus philippensis* (Lam.) Müll. Arg. by Furusawa et al.^37^. It recorded − 8.8 BE and was found interacting with AARs GLN493, GLN492, ARG403 and GLU406 of the spike protein. Bioavailability score of 0.55 was recorded by Kamalachalcone C, indicating it as a potent drug candidate (Fig. 1b, Supplementary File 2). Amentoflavone, a biflavone also recorded a lower BE of − 8.7 and showed H bond formation with AARs GLN493, SER494 and GLY496 of SARS-CoV-2 spike protein (Fig. 1b).

Recent studies also reported several PSM such as Pavetannin-C1 (BE − 11.1), Hesperidin (BE − 10.4), Cannabinoids (BE − 10.2), Cinnamtannin-B1 (BE − 10.2) with lower BE as a potential molecules to reduce the pathogenicity of SARS-CoV-2 by blocking the spike protein RBD and hACE2 interactions (Supplementary File 4).

### Human transmembrane serine protease (TMPRSS2)

**Figure 2:**
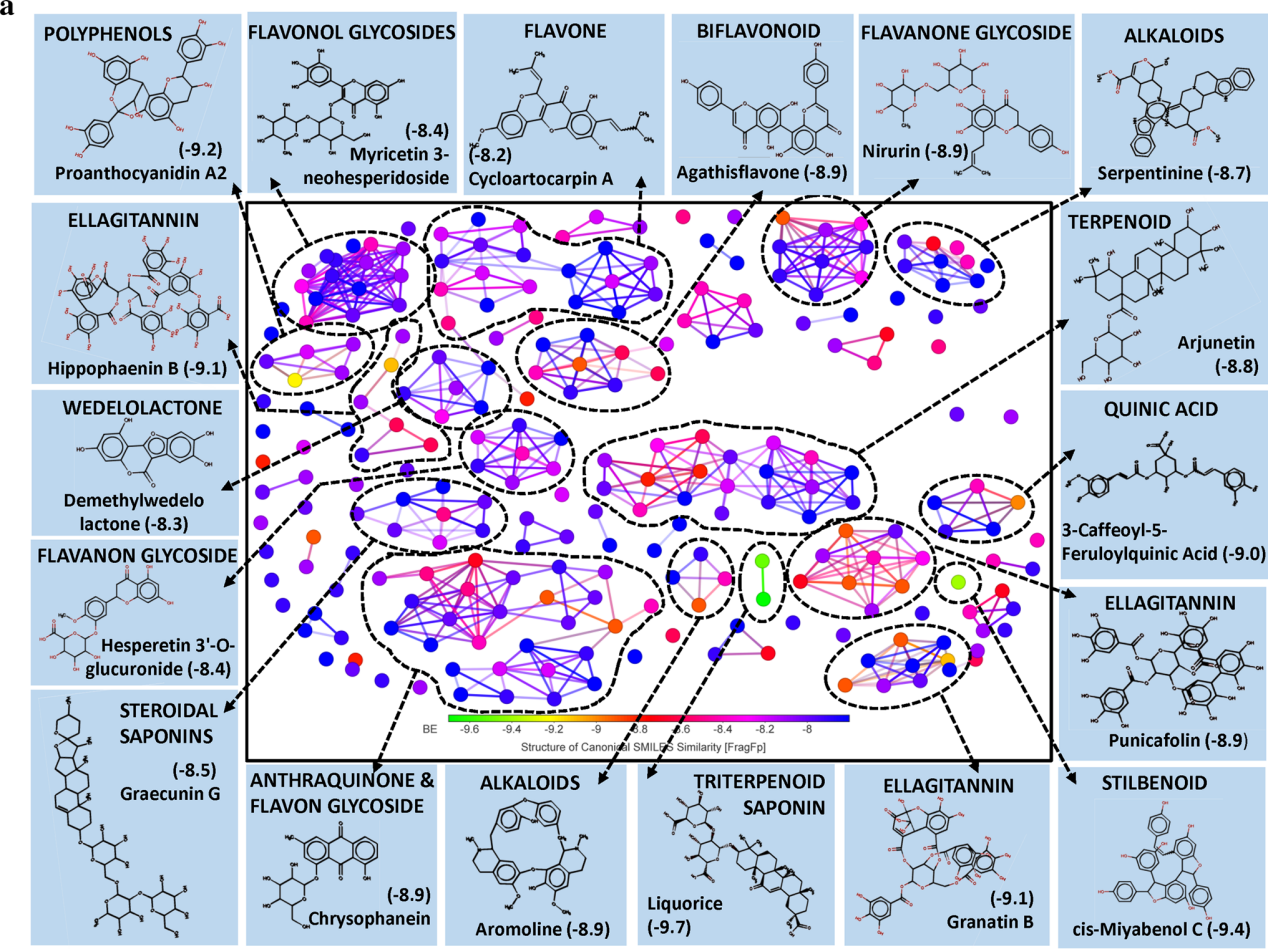

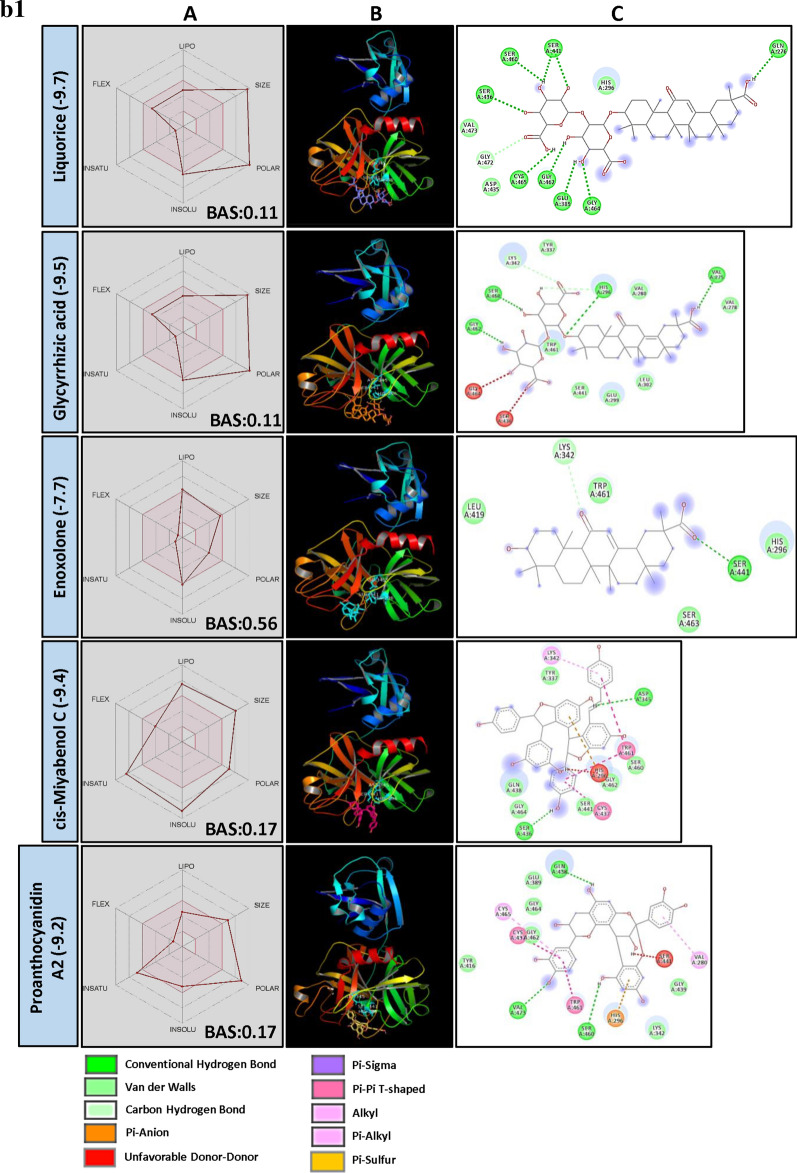

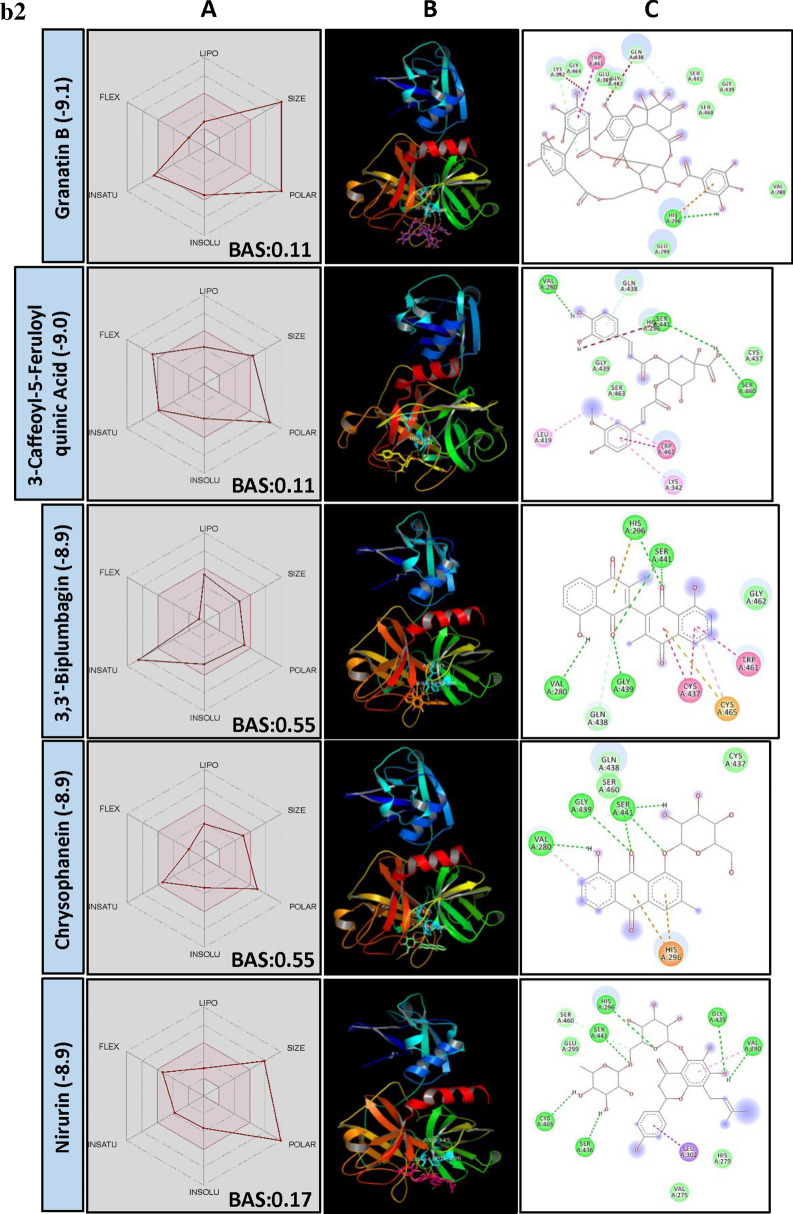
(**a**) Structural activity relationship: Correlation of canonical SMILES structure similarity (data points are joined by colored lines) and binding energy (represented in different color shades of data point) of selected plant secondary metabolites (PSM) evaluated against SARS-CoV-2 TMPRSS2 using Data Warrior software. Structurally similar molecules are grouped in dotted lines and a representative molecule with low binding energy (kcal/mol) (values in parenthesis) is represented in box. Among top ranked PSM studied, > 32% found belonging to Flavonoid glucoside and other major groups are ellagitannins and triterpenoids. (**b**) Data analysis of selected PSM against SARS-CoV-2 TMPRSS2. (**A**) Bioavailability radar chart representing lipophilicity (LIPO), Molecular weight (SIZE), Topological polar surface area (POLAR), Solubility (INSOLU), Flexibility (FLEX) and Saturation (INSATU) along with Bioavailability score (BAS) of selected molecules, (**B**) 3D visualization of protein–ligand interaction using PyMOL (selected amino acid residue of target site of protein are colored in cyan, and (**C**) 2D visualization of different types of interactions between ligand and target site of protein using Discovery Studio software (different types of interactions are represented in color codes).

TMPRSS2 plays a key role in priming spike protein of SARS-CoV-2. The cellular protease cleaves at S1/S2 and S′2 sites thereby facilitating the fusion of viral and host cell membrane^38^. Hence, reducing the protease activity of TMPRSS2 using PSM is considered as another potential way to manage COVID-19. In our study, structural similarity and BE correlation analysis indicated that a large number of flavonoid glucoside (> 32%) were found interacting with the target site of TMPRSS2, followed by ellagitannins and triterpenoids. Interestingly, two triterpenoid saponins (Liquorice and Glycyrrhizic acid) and a stilbenoid (Cis-Miyabenol C) recorded the lowest BE (Fig. 2a). The data obtained from this study indicated the possibilities of developing flavonoid glycoside or triterpenoid based drug molecules targeting human TMPRSS2 which process SARS-CoV-2 spike protein and facilitate the entry of the virus into the host cell.

In our study, a large number of flavonoid glycosides were found efficiently binding to the target site of protein with lower BE. Similarly, in most of the earlier in vitro studies, flavonoid glycosides are proved to be better candidates for enzyme inhibition activity related to health benefits. However, significant concern about flavonoid glycosides is their bioavailability. Upon consumption, at the small intestine, they absorbed mostly in their aglycon form at the cellular level. Absorption of glycosidic form mainly depends on their number of sugar moiety. Through many research studies, it was confirmed that flavonoid glycosides are converted to aglycones by gut microbes and decomposed to yield two different phenolic products. Whereas, catechin and procyanidins are bio-transformed into 5-(hydroxyphenyl)-γ-valerolactone found in blood plasma and urine as sulfate and glucuronide metabolites^39^. However, several earlier studies supported the hypothesis that glycosidic forms are more bioavailable and resistant to microbial degradation. Also, they can deliver aglycone moiety better when administrated as glycosidic form. Participation of active uptake of flavonoid glycosides by enterocytes was researched in detail. During this process, flavonoid glycosides are converted into aglycones by membrane-bound beta-glucosidase (reviewed by Kumar and Pandey)^40^. Here, Agatisflavone, a bioflavonoid recorded a BE of − 8.9 and found interacting with catalytic site AARs SER441 of TMPRSS2 forming hydrogen bonds. Nirurin, a prenylated flavonone glycoside found in *Phyllanthus niruri* L. recorded a BE of − 8.9 and showed H bond formation with HIS296 and SER441 of TMPRSS2 catalytic site and with VAL290, SER436, GLY439 and CYS465 in close vicinity of the catalytic site, presenting itself as a strong irreversible inhibitor of TMPRSS2 (Fig. 2b, Supplementary File 2). Following to this, Naringin, a flavanone-7-O-glycoside recorded BE of − 8.3 and showed similar interaction with AARs of TMPRSS2 (Supplementary File 2). All these molecules are low in their BAS and their physicochemical characters are not favourable as per Lipinski’s rule (Supplementary File 3).

Though the flavonoids are dominating the group in numbers, the least BE was recorded by two triterpenoid saponins, i.e., Liquorice (− 9.7) and Glycyrrhizic acid (− 9.5) followed by a stilbenoid Cis-Miyabenol (− 9.4). Here, Liquorice was found forming H bond with SER441 of TMPRSS2 catalytic site AAR through glucose moiety. Similarly, Glycyrrhizic acid also showed H bond formation with HIS296 of TMPRSS2 through oxygen involved in a glycosidic bond. Liquorice and Glycyrrhizic acid are isomers, triterpenoid glycosides obtained from roots of *Glycyrrhiza glabra* L. (Liquorice). Roots of this plant are traditionally used to alleviate jaundice, gastritis and bronchitis. Gancao, a Chinese herbal decoction of dried plant roots and stem, contains 3.63–13.06% Glycyrrhizin and well known for their therapeutic properties, including antiviral^41^. Glycyrrhizic acid was reported as an active component from the roots of *G. glabra* which inhibited the growth and cytopathology of both DNA and RNA virus viz., Herpes simplex type I, Newcastle disease virus, Vesicular stomatitis virus and Polio type I virus without affecting host cell activity and replication. Research regarding the bioavailability of Glycyrrhizic acid revealed that after oral administration, only bio-transformed Glycyrrhetic acid was detected in plasma at higher concentration^42,43^. It is because of the complete biotransformation of Glycyrrhizic acid to Glycyrrhetic acid by the activity of gut microbes. Hence, to know the capability of aglycon form, i.e., Glycyrrhetic acid (Enoxolone, not present in main PSM library) was docked and found it also form H bond with SER441 of TMPRSS2, but the BE was reduced to − 7.7 (Fig. 2b).

Cis-Miyabenol C, a stilbenoid (resveratrol timer) found in *Foeniculum vulgare* Mill. (fennel). Stilbenoids are characterized by two phenyl group linked by a transethane bond and reported to exhibit a wide range of biological activities and pharmacological properties^44^. In the present study, Cis-Miyabenol C recorded a lower BE of − 9.4 and found interacting with catalytic site AAR of TMPRSS2, i.e., ASP345 through H bond (Fig. 2b, Supplementary File 2). This compound was low in GI absorption, highly lipophilic, insoluble in water and found violating Lipinski's rule of drug-likeness. The low bioavailability of stilbenoids is mainly due to their rapid, extensive metabolism in the intestine and liver during and after absorption giving rise to a lower level of the free parent compound^45^.

Chrysophenols (Anthraquinones) are anthracene derivatives, and structurally they are tricyclic aromatic quinones with two ketone group attached to the central benzene ring. Here, two anthraquinone glycosides, Chrysophanein and Rhein-8-glycoside recorded lower BE of − 8.9 and − 8.9, respectively, which was lower than their structurally related flavonoid glycosides, Baicalein 6-glucoside (BE − 8.7) and Luteolin 3′-xyloside (BE − 8.5). Chrysophaenein showed H bond with SER441, an AAR of the catalytic site of TMPRSS2 and VAL280 and GLY439 in the catalytic pocket. Chrysophaenein passes all the parameters of Lipinski's rule with 0.55 BAS (Fig. 2b, Supplementary Files 2 and 3) proving itself as a better drug candidate to inhibit the activity of TMPRSS2.

Plumbagin is a naphthoquinone derivative from the roots of *Plumbago zeylanica* L. and well studied for its anti-cancerous property^46,47^. In our study, 3,3′-Biplumbagin recorded BE of − 8.9 and found interacting with HIS296 and SER441 of the catalytic site of TMPRSS2 and with VAL280 and GLY439 AARs in the close vicinity, showing its strong affinity towards the catalytic domain of TMPRSS2. Also, it recorded higher BAS of 0.55 and found fulfilling all necessary physicochemical characters for a drug-like molecule as per Lipinski's rule (Fig. 2b, Supplementary File 2 and 3).

Quinic acid is a carboxylated cyclohexanepolyol that is found in several plants like coffee, tomato, carrot, etc. and exists either in free form or as esters^48^. Quinic acid is a starting compound used to synthesise "Tamiflu", an anti-viral drug used to treat Influenza A and B virus^49^. Derivatives of Quinic acid were reported to be anti-viral in nature against Human immunodeficiency virus (HIV), Hepatitis B virus (HBV), Herpes simplex virus 1 and Dengue virus^50–53^. 3-Caffeoyl-5-feruloylquinic acid, in our study, recorded − 9.0 BE and found interacting with SER441 of TMPRSS2 through H bonding. Following this, structurally similar 3-O-Caffeoyloleanolic acid also recorded BE of − 8.7. Both the molecules recorded low BAS of 0.11 and violated Lipinski's rule of drug-likeness (Fig. 2b, Supplementary File 2). Studies of Gonthier et al.^54^ showed that chlorogenic acids, the esters of caffic and quinic acid are poorly bioavailable. However, their microbial bio-transformed metabolites such as m-Coumaric acid and derivatives of phenylproponic, benzoic and hippuric acid were found in higher concentration after oral administration of chlorogenic acids. The anti-viral activity of bio-transformed molecules is yet to be studied.

In the present study, group of bulky Ellagitannins such as Terflavin A, Terflavin B, Punicalin, Strictinin, Pedunculagin, Punicafolin, Tellimagradin I, Tercatain, Emblicanin A, Phyllanemblinin B, etc. recorded lower binding efficiency indicating their capability to inhibit the activity of TMPRSS2 enzyme. Similarly, Granatin B, an ellagitannin commonly found in the pericarp of *Punica granatum* L. recorded lower BE of − 9.1 followed by Granatin A (− 8.9). Granatin B recorded H bond formation with HIS296 of TMPRSS2. Bioavailability score of Granatin B was 0.11 and found violating Lipinski’s rule (Fig. 2b, Supplementary Files 2 and 3). However, they are all high molecular weight, and according to previous studies, they are poorly/ not bioavailable. Further, they hydrolyze into Ellagic acid and their complex derivatives in the intestine and can cross the gastrointestinal barrier into the bloodstream^55^. At the same time, gut microflora are reported to convert Ellagitannins to Urolithins, an anti-cancerous compounds^56,57^. Bioavailability of Ellagitannins metabolites was successfully proved in humans in the form of Ellagic acid in blood plasma^58^. Ellagic acid consists of a hydrophilic domain made up of four phenolic groups and two lactones, and a lipophilic domain made up of four rings. These domains, particularly the hydrophilic, which can H bond and accept an electron, thus determining a structural activity relationship. In our experiments, Ellagitannins derivatives 3′-O-Methyl ellagic acid 4-xyloside recorded BE of − 8.1 against TMPRSS2 (Supplementary File 2), indicating the possibilities of Ellagitannin metabolized products function as TMPRSS2 inhibitors.

Molecules such as Geniposide (BE − 14.6), Cytidine-5′-diphosphocholine (BE − 13.9), Durumolide K (BE − 13.2) and 5′-methoxyhydnocarpin D (BE − 13.5) were reported to be effective molecules of plant origin which can bind to active site of human TMPRSS2 and interfere with the viral spike protein priming activity (Supplementary File 4). However, with respect to TMPRSS2 activity inhibition, competitive inhibitors are preferred over uncompetitive/noncompetitive inhibitors as TMPRSS2 activity is an essential component of cellular structure and function.

### SARS-CoV-2 Main Protease (M^pro^)

The key enzyme in proteolytic processing of SARS-CoV-2 replication is M^pro^. It is initially released by the auto-cleavage of pp1a and pp1ab. Then M^pro^, in turn, cleaves pp1a and pp1ab to release functional proteins necessary for viral replication^59^. Any PSM binding to the AARs of the catalytic site or pocket with H bonds and other interactions with lower BE may interfere with the viral replication process in host cell, thereby reducing the severity of the COVID-19.

**Figure 3:**
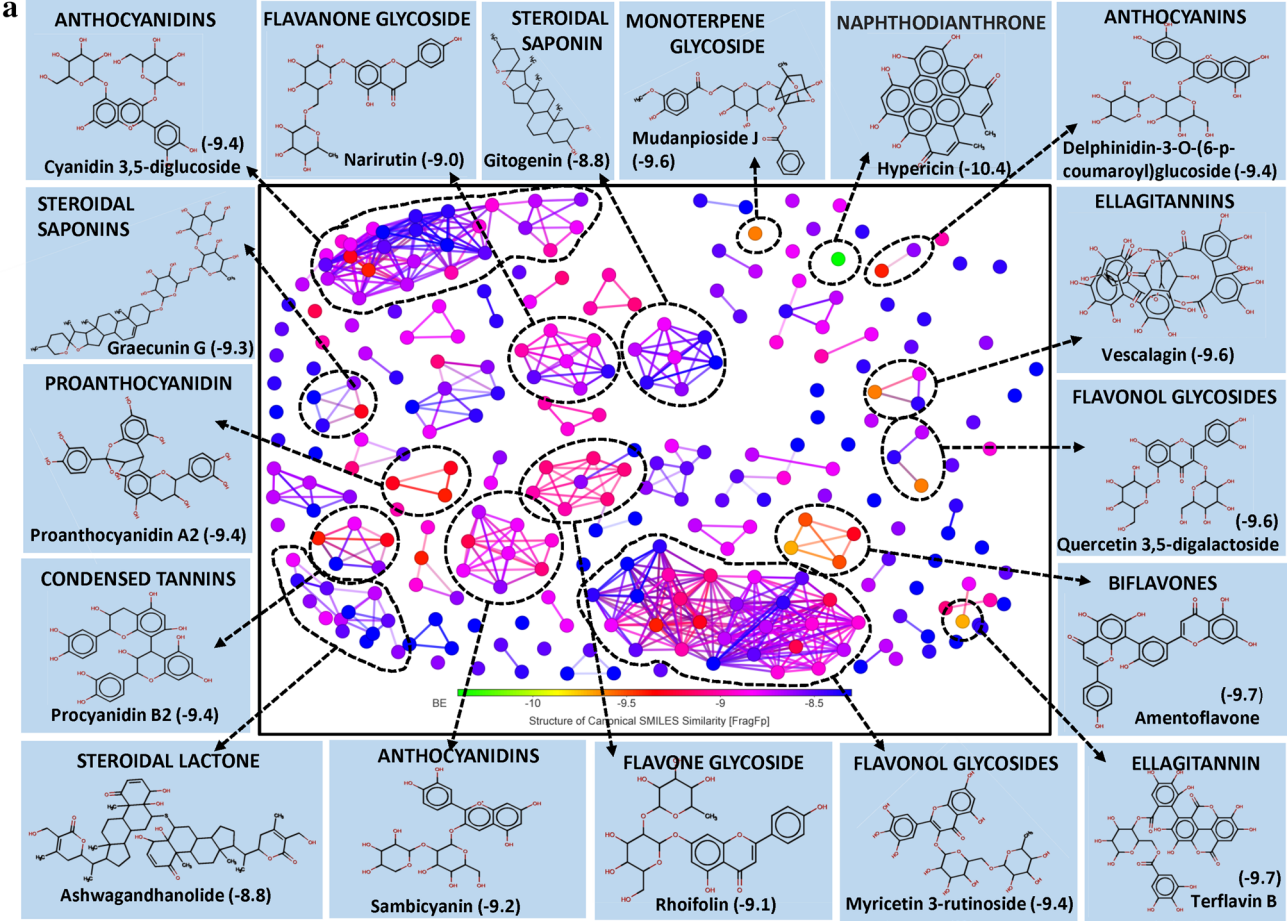

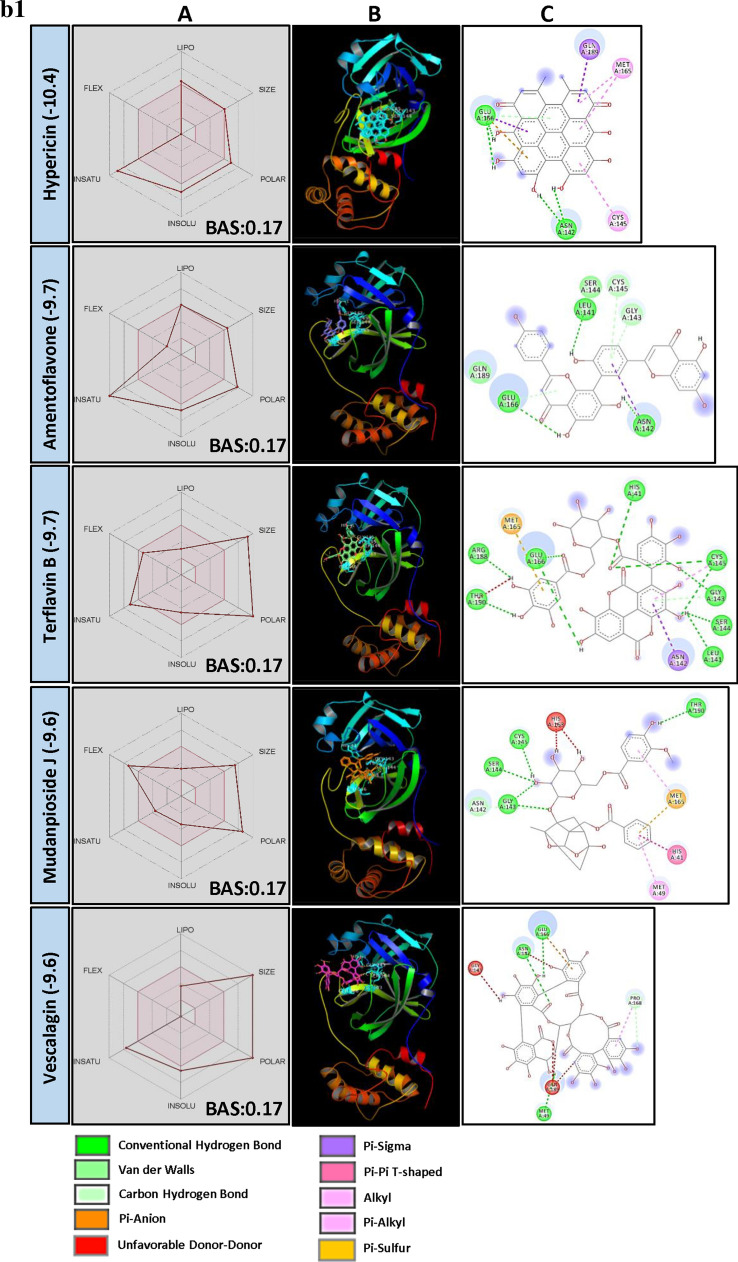

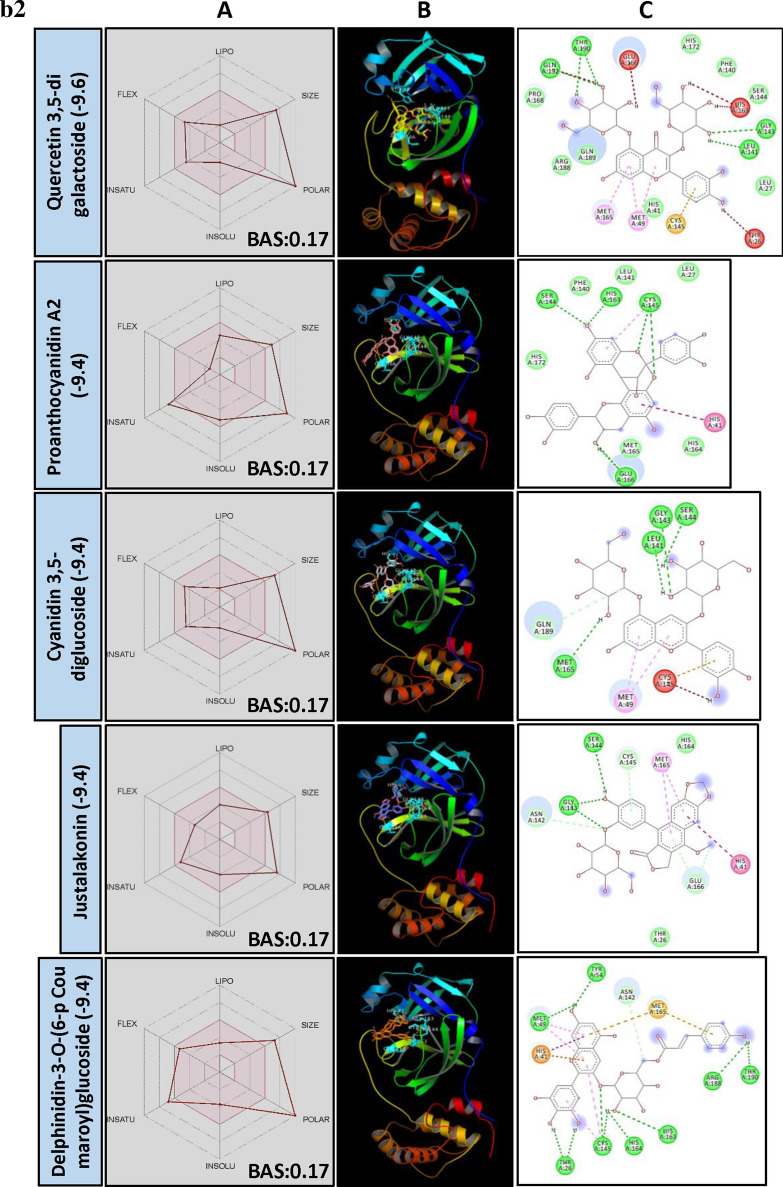
(**a**) Structural activity relationship: Correlation of canonical SMILES structure similarity (data points are joined by colored lines) and binding energy (represented in different color shades of data point) of selected plant secondary metabolites (PSM) evaluated against SARS-CoV-2 M^pro^ using Data Warrior software. Structurally similar molecules are grouped in dotted lines and a representative molecule with low binding energy (kcal/mol) (values in parenthesis) is represented in box. In this case, flavonol glycosides (> 16%) and Anthocyanidine (> 16%) are the largest group of PSM among the top ranked 250 PSM. (**b**) Data analysis of selected PSM against SARS-CoV-2 M^pro^. (**A**) Bioavailability radar chart representing lipophilicity (LIPO), Molecular weight (SIZE), Topological polar surface area (POLAR), Solubility (INSOLU), Flexibility (FLEX) and Saturation (INSATU) along with Bioavailability score (BAS) of selected molecules, (**B**) 3D visualization of protein–ligand interaction using PyMOL (selected amino acid residue of target site of protein are colored in cyan, and (**C**) 2D visualization of different types of interactions between ligand and target site of protein using Discovery Studio software (different types of interactions are represented in color codes).

When BE and canonical SMILES structural similarity of top 250 PSM were analyzed, it was observed that molecules from two major groups, i.e., Flavonol glycosides and Anthocyanidine were dominating with > 16% and > 16% PSM, respectively. Other flavonoids and triterpenes also recorded promising results (Fig. 3a). But, the least BE was recorded by Hypericin, a naphthodianthrone and Amentoflavone, a biflavonoid.

Hypericin, a naturally occurring chromophore “Naphthodianthrone” compound, derivative of anthraquinone found in common St. Johnswort (*Hypericum* species) and in some fungi. *Hypericum perforatum* L., a source of Hypericin has been used as folk medicine. Also, Hypericin is reported as anti-depressive, anti-tumor, anti-viral, antineoplastic, etc.^60^. Here, Hypericin recorded least BE of − 10.4. Further, it was found forming H bond with GLU166 residue in catalytic sites of M^pro^ (Fig. 3b, Supplementary File 2). Physicochemical parameters of Hypericin showed that it is poorly soluble in water and violates Lipinski's rule of drug-likeness (Fig. 3b, Supplementary File 2) with 0.17 BAS. It is a naturally occurring photosensitizer reported to accumulate in tumour cells and upon illuminating release Reactive oxygen species (ROS) killing the cancerous cells^61^. Recent in silico studies revealed its potential to bind SARS-CoV-2 spike protein^62^. Additionally, its anti-viral properties against Bronchitis virus^63^, Hepatitis C virus^64^ and Human Coronavirus Oc43^65^ represent it as a potential anti-viral drug material.

Sotetsuflavone, a biflavonoid from *Dacrydium balansae* Brongn. & Gris (Gymnosperm) was found to be the most potent inhibitor of Dengue virus NS5 RdRp with IC_50_ of 0.16 µM, among 23 biflavonids screened. Further, their enzyme inhibitory activity was related to the number and position of methyl groups on the biflavonoid moiety and the degree of oxygenation on flavonoid monomers^66^. In our studies, Amentoflavone recorded least of − 9.7 BE against M^pro^ and found interacting with target AAR by forming H bonds with GLU166 and other residues in the vicinity of catalytic site (Fig. 3b, Supplementary File 2). Similarly, other biflavonoids, both Ginkgetin and Isoginkgetin (derivatives of Amentoflavone from *Ginkgo biloba* L.) were recorded BE − 9.5 followed by Agatisflavone (BE − 9.3).

Flavonol glycosides are the most influential group of PSM found interacting with the target site of M^pro^. Flavonols are the class of flavonoids that has 3-hydroxyflavone backbone and present in a large group of plants. Lower BE of − 9.6 was recorded by Quercetin 3,5-diglucoside followed by Myricetin 3-rutinoside (BE − 9.4), Rutin (BE − 9.3), Kempferol (BE − 9.2), Myricetin 3-rhamnoside (BE − 9.2), and Robinetin 3-rutinoside (BE − 9.1) (Fig. 3b, Supplementary File 2). It was observed that flavonol with two glucose moieties recorded lower BE when compared to flavonol with one or three glucose moieties. With regards to bioavailability, Graefe et al.^67^ reported the heights of 2.3 and 2.1 µg/ml of quercetin glucuronides in plasma of human volunteers after oral administration of onion and quercetin-4′-O Glucoside, respectively. Similarly, the peaks of 4.3 and 4.0 µg/ml were recorded for buckwheat tea and rutin oral administration. The authors hypothesized that quercetin-4′-O glucoside and quercetin-3-O glucoside absorption might happen at a different site of GI tract and depend on the number of the sugar moiety. Further, plant matrix may have an additional effect on GI absorption of these flavonol glycosides.

Proanthocyanidins are a class of oligomeric flavonoids (condensed tannins) found in a variety of plants. They are formed by the condensation of catechin and epicatechin units. Studies on Proanthocyanidins reveal its poor bioavailability, its monomers viz., ( +)-catechin and (−)-epicatechin have been previously reported to cross GI track in its monomeric form or as conjugated metabolites^35^. Further, its dimers are also reported to present in plasma and urine after consuming food and beverages rich in Proanthocyanidins^68,69^. In our studies, Proanthocanidin A2 and Proanthocanidin B2 recorded least of − 9.4 BE followed by Procyanidine B1 (BE − 9.3). Further, their monomers/ dimers/ conjugates viz., (-)-Gallocatechin gallate (BE − 9.1), Epicatechin-3-gallate (BE − 9.0), Gallocatechin-(4alpha- > 8)-epigallocatechin (BE − 8.6) were found effective in the binding target site of SARS-CoV-2 M^pro^ (Supplementary File 2). Glycosidic anthocyanidin (anthocyanins), water-soluble pigments found in many fruits like grapes, bilberry, raspberry, blackberry, cherry, blueberry, etc. Here, Cyanidine-3,5, diglucoside recorded − 9.4 BE followed by Penodine 3,5 diglucoside (BE − 9.3). Further, H bond formation between Cyanidine-3,5, diglucoside and AARs GLY143 and SER144 of the catalytic site of M^pro^ was observed. It recorded a BAS of 0.17 and didn’t fulfill the criteria of a drug-like molecule as per Lipinski's rule (Fig. 3b, Supplementary File 2). Bioavailability of Cyanidine-3-glucoside was studied in human trials by Czank et al.^70^ following the isotopically labeled compound. They found radioactivity in plasma, urine and breathe, indicating the absorption of the labeled compound. However, the study didn’t reveal the existence of bioactive metabolites; it may be in degraded forms of the parental compound.

Terflavin B, an ellagitannin (found in *Terminalia chebula* Retz. and *T. catappa* L.) recorded BE of − 9.7. Also, it was found forming H bonds with AARs HIS41, GLY143, SER144, CYS145 and GLU166 present in the catalytic site of SARS-CoV-2 M^pro^. It is water-soluble, low in GI absorption and violates Lipinski's rule (Fig. 3b, Supplementary File 2). Aqueous extracts of *T. chebula* were reported to be inhibitory to Hepatites B virus infection in Hep G 2.2.15 cells^71^. Not much research studies on their bioavailability and anti-viral properties have been done with purified Terflavin B. Vescalagin, and Castalagin are ellagitannins found in oak and chestnut wood^72,73^. They are water-soluble, high oxidizable and astringent^74^. Here, Vescalagin recorded − 9.6 BE and found interacted with GLU166 residue of M^pro^ catalytic site through H bond, and Castalagin showed − 8.8 BE (Fig. 3b, Supplementary File 2). Vilhelmova et al.^75^ demonstrated the anti-viral activity of Vescalagin and Castalagin against Herpes simplex virus type I and II. Also, these PSM synergistically inhibited the multiplication of test virus along with anti-viral compound Acyclovir.

Mudanpioside J is a monoterpene glucoside identified in *Paeonia delavayi* Franch. (Chinese medicinal plant), which was found inhibitory to Influenza virus neuraminidase^76^. In our studies, Mudanpioside J recorded BE of − 9.6 and showed strong interaction with GLY143, SER144 and CYS145 residues of M^pro^ catalytic site through H bonds (Fig. 3b, Supplementary File 2). Studies on Pharmacokinetics properties of monoterpene glycosides are limited. Paeoniflorin and Albiflorin, Mudanpioside J related compounds were reported as low in oral bioavailability due to their poor membrane permeability and gut microbes-induced metabolism^77,78^. SwissADME analysis of Mudanpioside J also indicated its P-glycoprotein substrate nature and violation of Lipinski’s rule with 0.17 BAS (Supplementary File 3).

Lignans were reported for their wide biological activities, including anti-viral against Hepatitis B, Hepatitis C, Herpes simplex virus type 1 and 2, Epstein-Barr virus and Cytomegalovirus^79,80^. Justalakonin is an Arylnaphthalene lignan glycoside isolated from *Justicia purpurea* L. In the present study, it recorded BE of − 9.4 and showed interaction with AARs GLY143 and SER144 of the catalytic site of M^pro^ through H bonding (Fig. 3b, Supplementary File 2). Physicochemical characterization revealed it was found violating Lipinski's rule with 0.17 BAS. Regarding its metabolism and bioavailability, not much research studies have been performed earlier. Similarly, structurally related PSM, Elenoside (BE − 8.9), Patentiflorin B (BE − 8.9) and Patentiflorin A (BE − 8.6) also recorded lower BE against M^pro^ (Supplementary File 2).

As per recent computational studies, molecules like Theaflavin-3–3′- digallate (BE − 12.4), Delphinidin 3,5-diglucoside (BE − 12.2) and Rutin (BE − 11.3) were reported as potential inhibitors of SARS-CoV-2 M^pro^ with lowest BE and predicted to interfere with the process of viral replication within host cell (Supplementary File 4). However, bioavailability and biotransformation of such PSM are need to be studied in detail.

### SARS-CoV-2 RNA-dependent RNA polymerase (RdRp)

**Figure 4:**
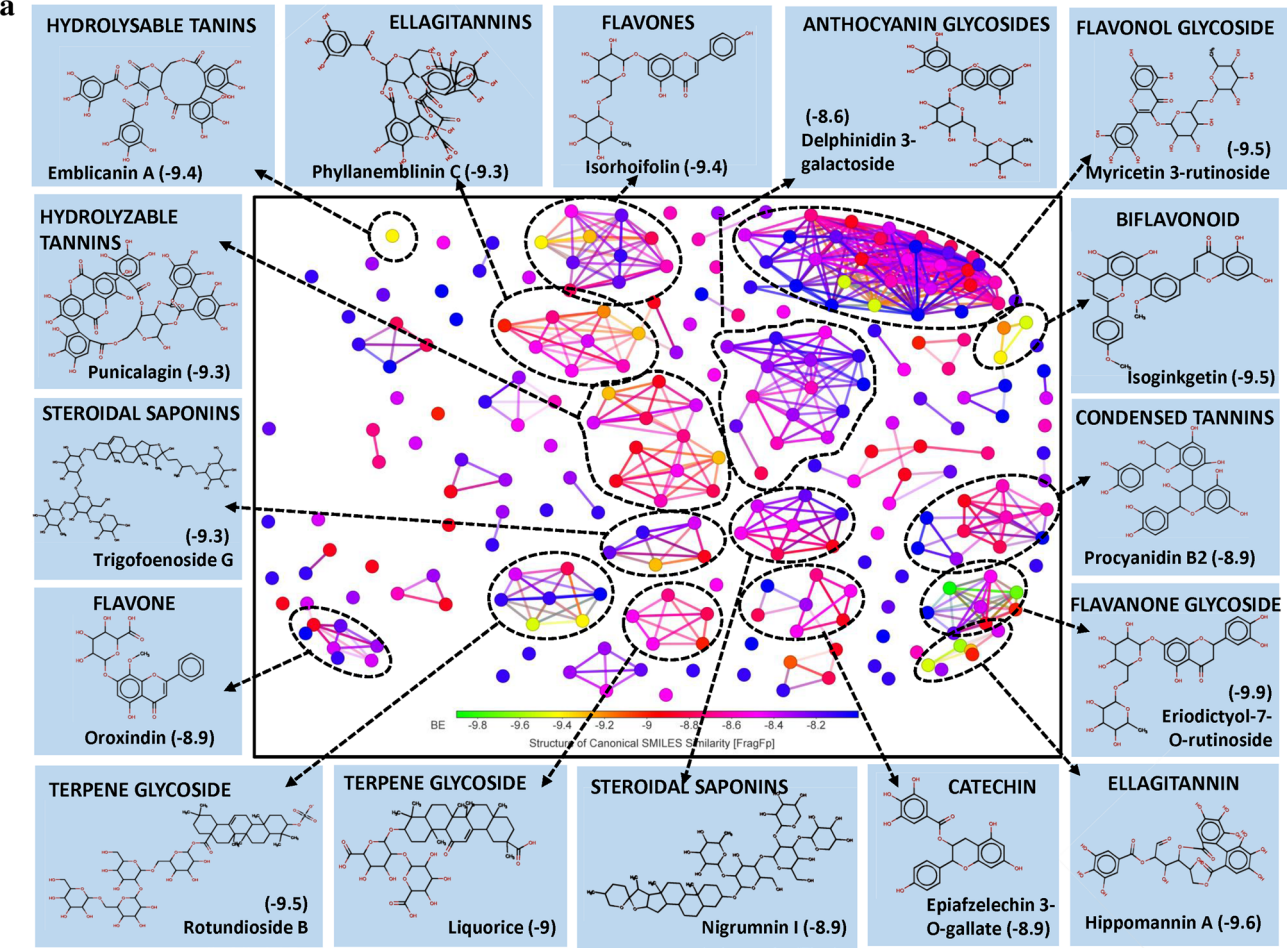

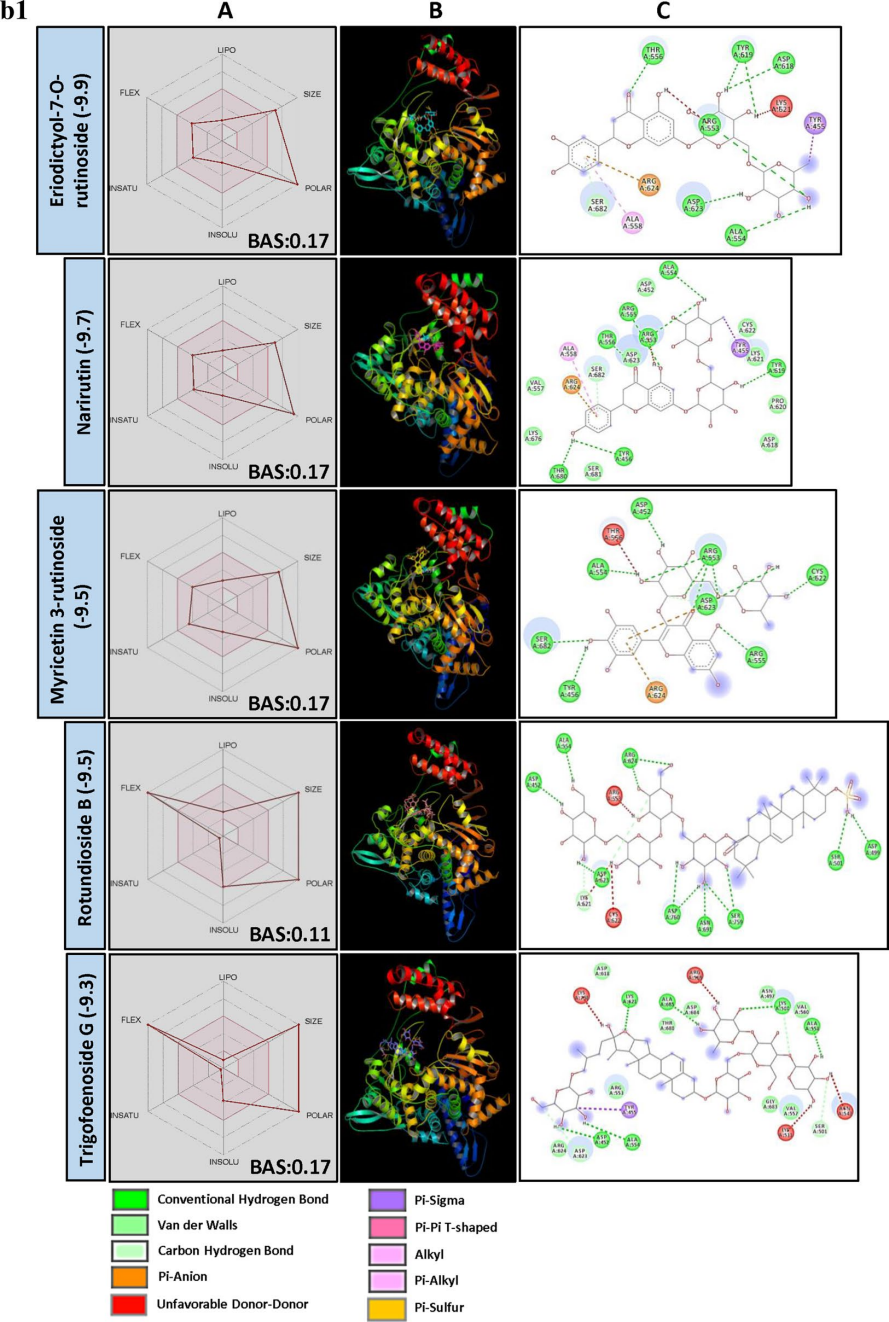

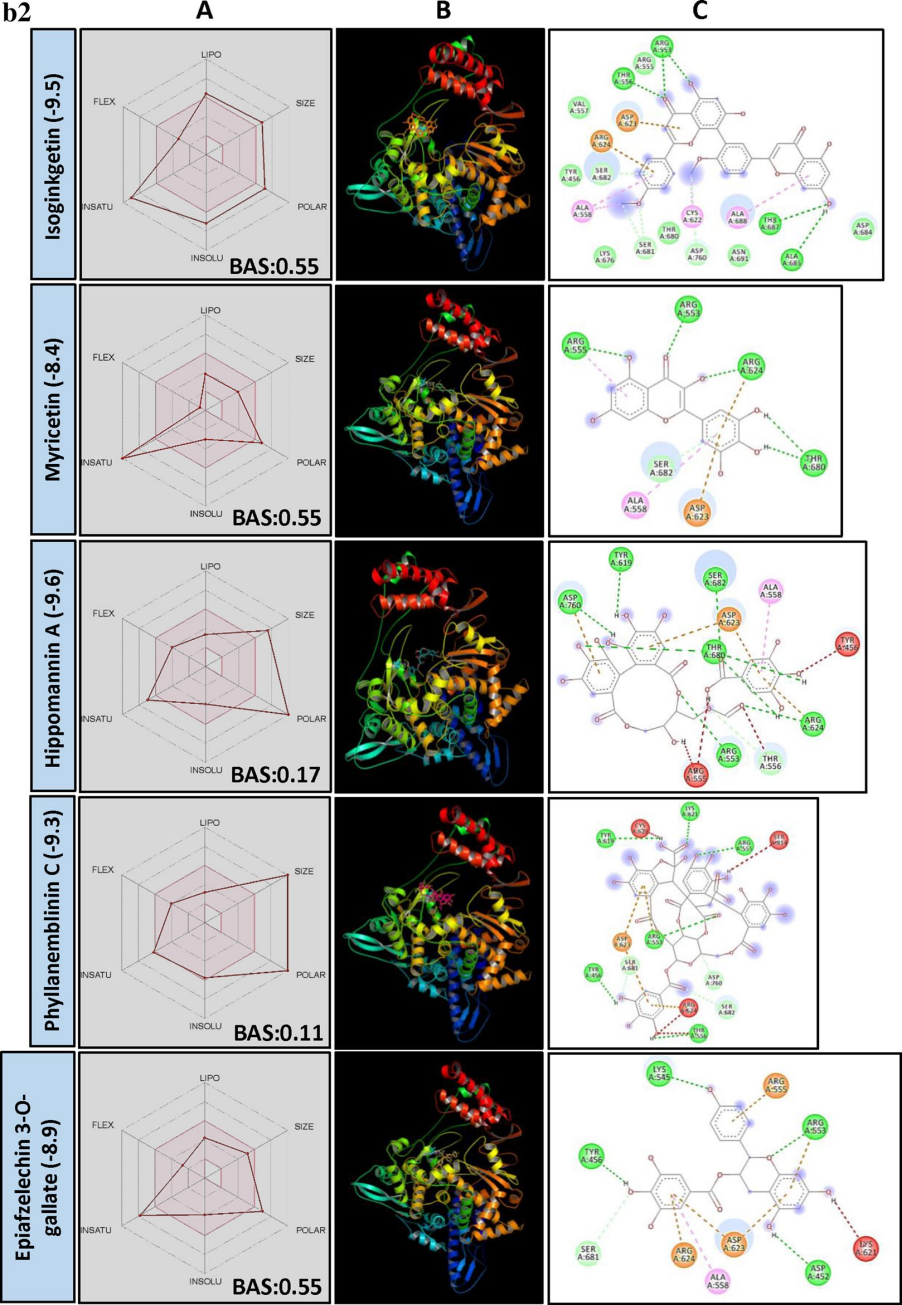
(**a**) Structural activity relationship: Correlation of canonical SMILES structure similarity (data points are joined by colored lines) and binding energy (represented in different color shades of data point) of selected plant secondary metabolites (PSM) evaluated against SARS-CoV-2 RdRp using Data Warrior software. Structurally similar molecules are grouped in dotted lines and a representative molecule with low binding energy (kcal/mol) (values in parenthesis) is represented in box. Here, large number of falvonol glycoside (> 13%) followed by hydrolysable tannins, anthocyanins and triterpenes are the major group of PSM found interacting with target site of SARS-CoV-2 RdRp. (**b**) Data analysis of selected PSM against SARS-CoV-2 RdRp. (**A**) Bioavailability radar chart representing lipophilicity (LIPO), Molecular weight (SIZE), Topological polar surface area (POLAR), Solubility (INSOLU), Flexibility (FLEX) and Saturation (INSATU) along with Bioavailability score (BAS) of selected molecules, (**B**) 3D visualization of protein–ligand interaction using PyMOL (selected amino acid residue of target site of protein are colored in cyan, and (**C**) 2D visualization of different types of interactions between ligand and target site of protein using Discovery Studio software (different types of interactions are represented in color codes).

The central component of coronaviral replication/ transcription machinery is RNA-dependent RNA polymerase (RdRp, also named nsp12) that constructs copies of its RNA genome playing the key role in replication and transcription of SARS-CoV-2 in the host cell^81,82^. Because of its high sequence and structural conservation, it remains the target of choice for the prophylactic or curative treatment of several viral diseases. Studies on structural activity relationship and BE of PSM revealed that a large number of flavonol glycoside (> 13%) followed by hydrolysable tannins, anthocyanins and triterpenes were found interacting with target site of RdRp with lower BE (Fig. 4a). Though the number of flavonol glycosides were high, the lowest BE was recorded by Erodictyol-7-O-glycoside and Narirutin belong to group flavanon glycosides. Interestingly, none of the PSM analyzed was found interacting with VAL557 of RdRp. However, they found forming H bond and another type of interactions with AARs in the catalytic pocket probably sterically hinders the substrate interaction with the catalytic site, thereby reducing the RdRp activity.

Eriodictyol-7-O-rutinosideis a flavanone glycoside commonly found in lemon^83^, also called as lemon or citrus flavonoid. It recorded − 9.9 BE and found interacting with ARG553, ALA554, THR556, ASP618, TRY619 and ASP623 in the active site of RdRp. Both glycone and aglycone moiety of this molecule were found involved in H bond formation with the target site of RdRp. Similarly, Narirutin, another flavanone glycoside which naturally presents in sweet oranges recorded lower BE of − 9.7 and a similar pattern of H bond formation with a catalytic pocket of RdRp was observed. Both molecules recorded BAS of 0.17, indicating their low bioavailability and found violating Lipinski’s rule (Fig. 4b, Supplementary File 2). Structurally similar compounds, Nirurin from *P. niruri* and Naringin from grapefruits also showed promising results with BE − 9.0 and − 8.9, respectively.

Myricetin 3-rutinoside is a flavonol glycoside isolated from *Chrysobalanus icaco* L.^84^ and other related plant species recorded − 9.5 BE. This molecule was found forming a large number of H bond with AARs ASP452, TYR456, ARG553, ALA554, ARG555, CYS622, ASP623 and SER682 showing its capability of strongly interacting with the target protein. Similarly, Kempferol (flavonol glycoside) also recorded − 9.5 BE. For both the compounds BAS was found to 0.11 (Fig. 4b, Supplementary File 2 and 3). Rhoifolin and Isorhoifolin are flavone glycoside reported from *Rhus succedanea* L.^85^ and several citrus plants and onions. It is reported as anti-oxidant, anti-inflammatory, anti-microbial, hepatoprotective and anti-cancerous (reviewed by Refaat et al.^86^). Though they recorded lower BE, Rhoifolin and Isorhoifolin were not interacting with AARs close to the active site of RdRp. Isoginkgetin, a biflavonoid from *G. biloba* recorded lower BE of − 9.5 followed by Agatisflavone (BE − 9.4) and ginkgetin (BE − 9.2). Isoginkgetin was found interacting with ARG553, THR556, THR687 and ALA685 of RdRp catalytic pocket. Compared to flavonoids glycosides, aglycons were frequently reported to be bioavailable. Hence, Myricetin, a monomeric aglyconic flavonoid, was analyzed and recorded BE − 8.4. Further, Myrcitin showed H bond with ARG553, ARG555, ARG624 and THR680 of RdRp active site, proving itself as a better candidate for developing drug targeting SARS-CoV-2 RdRp. Similarly, Quercitin glycosides were found promising, but quercetin was not able to appear in the list of top-ranked PSM.

Rotundioside B is a triterpenoid glycoside (Saponin) found in *Bupleurum rotundifolium* L.^87^ and reported as an anti-inflammatory and anti-proliferative^88,89^. Here, Rotundioside B recorded − 9.5 BE and formed H bond with ASP452, SER501, ALA554, ASP623, ARG624, ASN691, SER759 and ASP760 of RdRp catalytic pocket proving itself as a strong contender of an anti-viral drug. However, this molecule recorded poor BAS of 0.11 and violated Lipinski's rule of drug-likeness (Fig. 4b, Supplementary File 2). Similar to this, Ginsenoside Ro, a triterpene saponin from roots of *Panax ginseng* C.A. Meyer. and related plants recorded − 9.4 BE. Trigofoenoside G, a steroidal saponin from plants and seeds of *T. foenum-graecum* was found interacting with RdRp with − 9.3 BE and forms H bonds with ASP452, LYS500, ALA554, ALA558, LYS621 and ALA685 of catalytic pocket (Fig. 4b, Supplementary File 2). Following to this, Trigofoenoside F and Trigofoenoside A recorded − 8.9 and − 8.4 BE, respectively (Supplementary File 2).

Hippomanin A, an ellagitannin found in *Hippomane mancinella* L. is known for its toxic properties. It causes oropharyngeal and gastrointestinal tract lesions, hypotension and bradycardia^90,91^. Here, Hippomannin A recorded − 9.6 BE and forms H bond with ARG553, TYR619, ARG624, THR680, SER682 and ASP760 with target site of RdRp. A structurally similar molecule, Tellimagrandin I found widely in fruits, nuts and vegetables. Tellimargrandin was reported for its wide spectra of biological activities (reviewed by Zheng et al.^92^). Here, it recorded − 9.5 BE followed by Punigluconin (BE − 9.3). Other hydrolysable tannins viz., Emblicanin A and Phyllanemblinin C found in fruits of Indian Gooseberry (*Emblica officinalis* Gaertn.) were recorded lower BE of − 9.4 and − 9.3, respectively. As the above discussed hydrolysable tannins are highly water-soluble and larger in molecular weight, their bioavailability in their original form is a major concern. Some of the molecules, structure similar to their bio-transformed metabolites with higher bioavailability, like Ellagic acid (BE − 8.3), 3′-O-methyl ellagic acid-4-xyloside (BE − 8.4), and 3,3′-Di-O-methyl ellagic acid (BE − 8.2) also recorded promising results indicating the possible involvement of ellagitannins bio-transformed products in inhibiting RdRp activity thereby reducing the COVID-19 severity.

Recent studies also reported several PSM such as Cyanidin 3-(6″-manlonylglycoside) (BE − 11.5), Caftaric acid (BE − 10.6) and Chrysophanol 8-(6-galloylglucoside) (BE − 9.9) with lowest binding energy as a potential molecules to reduce the pathogenicity of SARS-CoV-2 by suppressing the activity of RdRp thereby reducing the viral multiplication capability inside host cell (Supplementary File 4).

## Conclusion

From the obtained results, it could be concluded that virtual screening of large number of PSM through molecular docking is a promising preliminary step towards developing an effective drug against a desired target protein/ enzyme by understanding their structure–activity relationship. Here, more than the BE of a PSM, its bioavailability also plays a crucial role in determining its biological activity under in vivo environmental conditions. Triterpenoid based PSM structures (Coagulins, Withanolides, Pseudojervine, Kamalachalcone, etc.) are hypothesized as potent drug molecules which can be used to block surface AARs of spike protein which interacts with hACE2, thereby preventing host cell recognition by SARS-CoV-2. In the case of TMPRSS2, M^pro^ and RdRp, molecules belong to flavonoid glycosides, biflavonoids, ellagitannins, anthocyanidins, triterpens, etc. (Table 1) can be explored. Though the large numbers of PSM were found violating Lipinski’s rule and recorded lower BAS, they can’t be ignored. Because several bio-transformed structure of these PSM are highly bioavailable and they may retain structural moiety of the parental compound. These bio-transformed molecules may further interact with the target site of a protein and exert similar results as observed in molecular docking studies. In our study, most of the potential anti-SARS-CoV-2 PSM were well studied for human consumption to manage various diseases and disorders previously. Also their controlled administration was proved to be non-toxic to humans. By considering the above facts, the possibilities of using these molecules along with the existing best practices to be explored immediately. Further, to streamline the large pools of PSM, they can be subjected for several in vitro and in vivo studies.

## Materials and methods

### Preparation of plant secondary metabolites library

To prepare PSM library, an extensive literature survey was conducted on selected plants and the general and species-specific PSM including Alkaloids, Phenolics and Terpenoids were listed. The 3D and 2D structures (SDF Files), and canonical SMILES of the selected PSM were retrieved from online databases such as PubChem (https://pubchem.ncbi.nlm.nih.gov) and ChemSpider (https://www.chemspider.com). The 2D structures were converted into 3D coordinates, and geometries were optimized by using Marvin Sketch (https://www.chemaxon.com/products/marvin/marvinsketch). As several PSM are present in multiple plant species, an approximate 6% duplication was allowed in the main PSM library. Additionally, PSM isomers were considered as separate ligands. All the files were coded and used for further studies.

### Target proteins

In the present study, we selected four target proteins, one from human (human transmembrane serine protease 2, TMPRSS2) and three from SARS-CoV-2 (spike protein, M^pro^ and RdRp). These selected enzymes/ proteins are well studied for their involvement in host cell recognition, membrane fusion, and viral replication in host cell, which are critical stages in determining viral pathogenicity. The crystal structure of SARS-CoV-2 spike receptor-binding domain bound with hACE2 (PDB ID: 6M0J) (2.45 Å) was retrieved from Research Collaboratory for Structural Bioinformatics (RCSB) Protein Data Bank (PDB) (RCSB, http://www.rcsb.org)^93^ and hACE2 was removed, processed and used for docking studies. The 14 amino acid residues (AARs) (THR415, ASN439, TYR449, TYR453, LEU455, PHE486, ASN487, TYR489, GLN493, GLN498, THR500, ASN501, GLY502 and TYR505) of spike protein that are key for binding hACE2^94^ were considered as active sites for molecular docking process. The TMPRSS2 sequence (NP_001128571.1) was retrieved from the National Center for Biotechnology Information (NCBI) protein database. The structure of TMPRSS2 was generated by using the SWISS-MODEL online server^95^. The structures were marked, superimposed and visualized by using Chimera^96^. Model 1 of our work and O15393 present in UniProt were found 100% similar. Three amino acid residues (HIS296, ASP345 and SER441) of catalytic site^97^ were considered as key residues of TMPRSS2 in the molecular docking process. The crystal structure of SARS-CoV-2 M^pro^ (PDB ID: 6LU7) (2.16 Å)^98^ was retrieved from RCSB-PDB and used for docking studies after processing. M^pro^ has three domains, and the active site is located between domain I and II. Here, CYS145-HIS41/SER144-HIS163 can act as a nucleophilic agent and GLY143 and GLU166 can form hydrogen bonds with “CO–NH-Cα-CO–NH-Cα” structure of the backbone of the substrate protein. These six residues were considered as the critical residues of M^pro^ in the molecular docking process^99^. The SARS-CoV-2 RdRp protein sequence (YP_009725307.1) was retrieved from the NCBI protein database. The structure of RdRp was generated by using the SWISS-MODEL online server^95^. The structure was marked, superimposed and visualized by using Chimera^96^. The amino acid residue VAL557 in motif F^82^ was considered as a critical residue of RdRp in the molecular docking process.

Removal of water molecules, metal ions, cofactors, and addition of charges and hydrogen atoms were done by UCSF Chimera tool^96^. Computing energy minimization and reconstruction of missing atoms and to perform stereo-chemical quality checks to come up at the best possible 3D structures were done through Discovery Studio software (Dassault Systèmes BIOVIA, Discovery Studio Modeling Environment, Version 3, San Diego: Dassault Systèmes, 2019).

### Molecular docking

The ligands were energy minimized by conjugate gradients optimization algorithm with total numbers of 200 steps performed as a default universal force field (UFF) parameters^100^. The capability of ligands to interact with the target site of selected proteins was studied following computational ligand-target docking approach. Molecular docking was carried out using PyRx, AutoDock Vina option based on scoring functions^101,102^. The least binding energy (BE, kcal/mol) conformation was considered as the most favourable docking pose. The interactions between ligand and protein were analyzed using PyMOL^103^ and Discovery Studio 3.5 (Accelrys Software Inc., San Diego, CA, USA).

### Structural activity relationship analysis

According to their BE, all the ligands were assigned ranking and aligned in ascending order. For the ease of the study, top 250 molecules (268 for M^pro^) were subjected to ligand structure similarity analysis (based on canonical SMILES) and BE using Data Warrior software (Version 5.2.1).

### Physicochemical properties and bioavailability of PSM

The drug-likeness and the physicochemical properties were studied using SwissADME (www.swissadme.ch). The canonical SMILES of the selected PSM were subjected to SwissADME analysis. The PSM were analyzed for their drug-likeness properties following Lipinski's rule of five^104^. The bioavailability radar charts obtained were analyzed for their drug like properties, i.e., lipophilicity (XLOGP3 between − 0.7 and + 5.0), Molecular weight (between 150 and 500 g/mol), Topological polar surface area (between 20 and 130 Å^2^), Solubility (log *S* not higher than 6), Flexibility (no more than 9 rotatable bonds) and Saturation (fraction of carbons in the sp^3^ hybridization not less than 0.25)^105^. The bioavailability score (BAS) for selected PSM was also recorded^106^.

## Supplementary information

Supplementary Information 1.

Supplementary Information 2.

Supplementary Information 3.

Supplementary Information 4.

Supplementary Information 5.

## Data Availability

The supporting data related to the manuscript is provided as Supplementary Files.
