# Supplementary material for: In silico studies evidenced the role of structurally diverse plant secondary metabolites in reducing SARS-CoV-2 pathogenesis
Source: Sci Rep. 2020 Nov 25;10:20584. doi: 10.1038/s41598-020-77602-0 (PMC7689506; doi:10.1038/s41598-020-77602-0)
Supplement: Supplementary file 1 — Supplementary Information 1. [file 41598_2020_77602_MOESM1_ESM.docx]

**Supplementary Information**

Title: In silico studies evidenced the role of structurally diverse plant secondary metabolites in reducing SARS-CoV-2 pathogenesis

Hariprasad Puttaswamy*, Hittanahallikoppal Gajendramurthy Gowtham, Monu Dinesh Ojha, Ajay Yadav, Gourav Choudhir, Vasantharaja Raguraman, Bhani Kongkham, Koushalya Selvaraju, Shazia Shareef, Priyanka Gehlot, Faiz Ahamed & Leena Chauhan

Centre for Rural Development and Technology

Indian Institute of Technology Delhi

New Delhi, Delhi – 110016 India

Supplementary Table 1: List of the plant secondary metabolites used in the present study

Supplementary Table 2a. List of the top tanked plant secondary metabolites with least binding energy against SARS-CoV-2 Spike protein

Supplementary Table 2b. List of the top tanked plant secondary metabolites with least binding energy against SARS-CoV-2 RdRp

Supplementary Table 2c. List of the top tanked plant secondary metabolites with least binding energy against SARS-CoV-2 TMPRSS2

Supplementary Table 2d. List of the top tanked plant secondary metabolites with least binding energy against SARS-CoV-2 Mpro

Supplementary Table 3. Physicochemical and Drug like properties of selected pant secondary metabolites

Supplementary Table 4. Binding energy recorded by various PSM against selected target proteins/enzymes aiming to reduce pathogenicity of SARS-CoV-2 pathogenesis from previous literature.
