# Supplementary material for: In silico studies evidenced the role of structurally diverse plant secondary metabolites in reducing SARS-CoV-2 pathogenesis
Source: Sci Rep. 2020 Nov 25;10:20584. doi: 10.1038/s41598-020-77602-0 (PMC7689506; doi:10.1038/s41598-020-77602-0)
Supplement: Supplementary file 5 — Supplementary Information 5. [file 41598_2020_77602_MOESM5_ESM.docx]

**Supplementary Table 4. Binding energy recorded by various PSM against selected target proteins/enzymes aiming to reduce pathogenicity of SARS-CoV-2 pathogenesis from previous literature.**

| **S. No.** | **Compound Name** | **Plant Name** | **Target**  **Protein**  **(PDB id)** | **Binding**  **Energy**  **(Kcal/Mol)** | **Reference** |
| --- | --- | --- | --- | --- | --- |
| **Target Protein: SARS-CoV-2 Main Protease (M^pro^**) | | | | | |
| 1 | Theaflavin-3-3′- digallate | *Camellia sinensis* | 6LU7 | -12.4 | Shivanika *et al*., 2020 |
| 2 | Delphinidin 3,5-diglucoside | *Aristotelia chilensis* | 6LU7 | -12.2 | Sharma and Shanavas, 2020 |
| 3 | Rutin | *Ruta graveolens* | 6LU7 | -11.3 | Shivanika *et al*., 2020 |
| 4 | Hypericin | *Hypericum perforatum* | 6LU7 | -11.1 | Shivanika *et al*., 2020 |
| 5 | Robustaflavone | Selaginella sellowii | 6LU7 | -10.9 | Shivanika *et al*., 2020 |
| 6 | (-)-Solenolide A | Briareum violaceum | 6LU7 | -10.8 | Shivanika *et al*., 2020 |
| 7 | Rhusflavone | *Rhus parviflora* | 6LU7 | -10.7 | Shivanika *et al*., 2020 |
| 8 | Ginkgetin | *Ginkgo biloba* | 6LU7 | -10.4 | Shivanika *et al*., 2020 |
| 9 | Rhinacanthin E | *Rhinacanthus nasutus* | 6LU7 | -10.4 | Shivanika *et al*., 2020 |
| 10 | Withanoside V | *Withania somnifera* | 6LU7 | -10.3 | Shree *et al*., 2020 |
| 11 | 3,5-Di-O-galloylshikimic acid | *Myrothamnus flabellifolius* | 6LU7 | -10.3 | Sharma and Shanavas, 2020 |
| 12 | Sorbarin | *Sorbaria stellipila* | 6LU7 | -10.0 | Shivanika *et al*., 2020 |
| 13 | Betulinic acid | *Betula pubescens* | 6LU7 | -9.9 | Shivanika *et al*., 2020 |
| 14 | Somniferine | *Withania somnifera* | 6LU7 | -9.6 | Shree *et al*., 2020 |
| 15 | Avicularin | *Polygonum aviculare* | 6LU7 | -9.6 | Sharma and Shanavas, 2020 |
| 16 | Peonidin-3-glucoside | *Vitis vinifera* | 6LU7 | -9.4 | Majumder and Mandal, 2020 |
| 17 | Kaempferol 3-O-β -rutinoside | *Rhamnus alaternus* | 6LU7 | -9.3 | Majumder and Mandal, 2020 |
| 18 | Scutellarein 7-glucoside | *Reseda luteola* | 6LU7 | -9.3 | Sharma and Shanavas, 2020 |
| 19 | Bonducellpin D | *Caesalpinia bonduc* | 6Y2F | -9.2 | Gurung *et al*., 2020 |
| 20 | 5,7-dimethoxyflavaN-4′-O-β-d-glucopyranoside | Viscum articulatum | 6Y2F | -9.2 | Gurung *et al*., 2020 |
| **Target Protein: SARS-CoV-2 RNA-Dependent RNA Polymerase (RdRp)** | | | | | |
| 1 | Cyanidin 3-(6”-manlonylglycoside) | *Cichorium intybus* | [6M71](http://xlink.rsc.org/?pdb=6M71) | −11.5 | Shawky, *et al*., 2020 |
| 2 | Caftaric acid | *Marrubium vulgare* | [6M71](http://xlink.rsc.org/?pdb=6M71) | −10.6 | Eman *et al*, 2020 |
| 3 | Chrysophanol 8-(6-galloylglucoside) | *Rumex dentatus* | 7BV2 | −9.9 | Alamri *et al*., 2020 |
| 4 | Luteolin 7-rutinoside | *Marrubium vulgare* | 7BV2 | −9.8 | Alamri *et al*, 2020 |
| 5 | Fenugreekine | *Trigonella foenum-graecum* | [6M71](http://xlink.rsc.org/?pdb=6M71) | −9.8 | Eman *et al*, 2020 |
| 6 | Isoorientin 6-O''-beta-D-glucopyranoside | *Tribulus terrestris* | [6M71](http://xlink.rsc.org/?pdb=6M71) | −9.7 | Eman *et al*, 2020 |
| 7 | Isoquercitrin | *Eruca sativa* | [6M71](http://xlink.rsc.org/?pdb=6M71) | −9.7 | Eman *et al*, 2020 |
| 8 | Cyanidin 3,5-diglucoside | *Hibiscus sabdariffa* | [6M71](http://xlink.rsc.org/?pdb=6M71) | −9.7 | Eman *et al*, 2020 |
| 9 | Apigenin-7-o-rutinoside | *Mtricaria chamomilla* | [6M71](http://xlink.rsc.org/?pdb=6M71) | -9.7 | Eman *et al*, 2020 |
| 10 | Glycyrrhizic acid | *Glycyrrhiza glabra* | [6M71](http://xlink.rsc.org/?pdb=6M71) | −9.6 | Eman *et al*, 2020 |
| 11 | Kaempferol-3-o-Glucuronide | *Medicago sativa* | [6M71](http://xlink.rsc.org/?pdb=6M71) | −9.5 | Eman *et al*, 2020 |
| 12 | Terrestric acid | *Tribulus terrestris* | [6M71](http://xlink.rsc.org/?pdb=6M71) | −9.5 | Eman *et al*, 2020 |
| 13 | luteolin 8-C-β-glucopyranoside | *Trigonella foenum-graecum* | [6M71](http://xlink.rsc.org/?pdb=6M71) | −9.4 | Eman *et al*, 2020 |
| 14 | Fraxicarboside A | [*Fraxinus oxycarba*](http://african-compounds.org/nanpdb/get_species_card/3126/) | [6M71](http://xlink.rsc.org/?pdb=6M71) | -9.4 | Eman *et al*, 2020 |
| 15 | Rhamnoliquiritin | *Glycyrrhiza glabra* | [6M71](http://xlink.rsc.org/?pdb=6M71) | −9.4 | Eman *et al*, 2020 |
| 16 | Apigenin 7-gentiobioside | *Artemisia judaica* | [6M71](http://xlink.rsc.org/?pdb=6M71) | −9.4 | Eman *et al*, 2020 |
| 17 | Rocymosin B | *Glycyrrhiza glabra* | [6M71](http://xlink.rsc.org/?pdb=6M71) | −9.3 | Eman *et al*, 2020 |
| 18 | Scropolioside D | *Scrophularia saharae* | [6M71](http://xlink.rsc.org/?pdb=6M71) | −9.3 | Eman *et al*, 2020 |
| 19 | Kaempferol 7-(6″-galloylglucoside) | *Acacia* | 7BV2 | −9.3 | Alamri *et al*, 2020 |
| 20 | Quercetin-3-gentiobioside | *Tribulus terrestris* | [6M71](http://xlink.rsc.org/?pdb=6M71) | −9.2 | Eman *et al*, 2020 |
| **Target Protein : SARS CoV-2 spike protein** | | | | | |
| 1. | Pavetannin-C1 | *Cinnamon* | 6LZG | -11.1 | Prasanth *et al*. (2020) |
| 2. | Hesperidin | *Citrus* spp.,  *Mentha* spp.,  *Linaria vulgaris* | 6VXX | -10.4 | Tallei *et al*. (2020) |
| 3. | Cannabinoids | *Cannabis* spp. | 6VXX | -10.2 | Tallei *et al*. (2020) |
| 4. | Cinnamtannin-B1 | *Cinnamon* spp. | 6LZG | -10.2 | Prasanth *et al*. (2020) |
| 5. | 6-Glucopyranosyl procyanidin B1 | *Cinnamon* spp. | 6LZG | -9.9 | Prasanth *et al*. (2020) |
| 6. | Pectolinarin | *Cirsium* spp., *Linaria vulgaris* | 6VXX | -9.8 | Tallei *et al*. (2020) |
| 7. | Epigallocatechin gallate | *Camellia sinensis,*  *Malus domestica,*  *Prunus domestica,*  *Allium cepa,* *Corylus avellana* | 6VXX | -9.8 | Tallei *et al*. (2020) |
| 8. | Procyanidin-B7 | *Cinnamon* | 6LZG | -9.6 | Prasanth *et al*. (2020) |
| 9. | Scopadulcic acid | – | 6VSB | -9.6 | Ubani *et al*. (2020) |
| 10. | Rhoifolin | *Rhus succedanea,*  *Citrus aurantium,*  *Citrus bergamia,*  *Citrus paradise,*  *Citrus limon,*  *Lablab purpureus,*  *Lycopersicon esculentum,*  *Cynara scolymus,*  *Musa* spp., *Vitis vinifera* | 6VXX | -9.5 | Tallei *et al*. (2020) |
| 11. | Proanthocyanidin-A2 | *Cinnamon* | 6LZG | -9.4 | Prasanth *et al*. (2020) |
| 12. | Baicalin | – | 6VSB | -9.4 | Ubani *et al*. (2020) |
| 13. | Sylibinin | – | 6VSB | -9.2 | Ubani *et al*. (2020) |
| 14. | Solanidine | – | 6VSB | -9.1 | Ubani *et al*. (2020) |
| 15. | Naringenin | – | 6VSB | -9.0 | Ubani *et al*. (2020) |
| 16. | Oleanane | – | 6VSB | -9.0 | Ubani *et al*. (2020) |
| 17. | Morin | *Maclura pomifera,* *Prunus dulcis,* *Chlorophora tinctoria, Psidium guajava* | 6VXX | -8.8 | Tallei *et al*. (2020) |
| 18. | Kaempferol 3-alpha-L-arabinofuranoside-7-rhamnoside | *Cinnamon*  spp. | 6LZG | -8.7 | Prasanth *et al*. (2020) |
| 19. | Tenuifolin | *Cinnamon*  spp. | 6LZG | -8.7 | Prasanth *et al*. (2020) |
| 20. | Kaempferol | *Brassica oleracea*  *Phaseolus vulgaris,*  *Camellia sinensis,*  *Spinacia oleracea,*  *Brassica oleracea* | 6VXX | -8.5 | Tallei *et al*. (2020) |
| **Target Protein: Human transmembrane serine protease (TMPRSS2)** | | | | | |
| 1 | Geniposide | *Rubiaceae* | 5CE1 | -14.6 | Rahman *et al*., 2020 |
| 2 | Cytidine-5’-diphosphocholine | *Rubiaceae* | 5CE1 | -13.9 | Rahman *et al*., 2020 |
| 3 | Durumolide K | *Rubiaceae* | 5CE1 | -13.2 | Rahman *et al*., 2020 |
| 4 | 5’-methoxyhydnocarpin D | *Rubiaceae* | 5CE1 | -13.5 | Rahman *et al*., 2020 |
| 5 | (-)Epicatechin-3-O-(3’-O-Methyl)Gallate | *Rubiaceae* | 5CE1 | -13.1 | Rahman *et al*., 2020 |
| 6 | Isogemichalcone B | *Artocarpusand Broussonetia* | 5CE1 | -13.0 | Rahman *et al*., 2020 |
| 7 | Neohesperidine | *Artocarpusand Broussonetia* | 5CE1 | -12.7 | Rahman *et al*., 2020 |
| 8 | Fuscaxanthone A | *Garciniaspp.* | 5CE1 | -12.3 | Rahman *et al*., 2020 |
| 9 | Orthosiphonone D | *Orthosiphon stamineus* | 5CE1 | -12.0 | Rahman *et al*., 2020 |
| 10 | Myricitrin | *Orthosiphon stamineus* | 5CE1 | -11.5 | Rahman *et al*., 2020 |
| 11 | Naringin | *Orthosiphon stamineus* | 5CE1 | -11.2 | Rahman *et al*., 2020 |
| 12 | 7-hydroxy-14-deoxywithanolide U | *Withania somnifera* | 5CE1 | -11.1 | Rahman *et al*., 2020 |
| 13 | 6S,9R-roseoside | *Ocimimum* spp. | 5CE1 | -11.1 | Rahman *et al*., 2020 |
| 14 | Quercitrin | *Ocimimum* spp. | 5CE1 | -10.8 | Rahman *et al*., 2020 |
| 15 | Icariin | *Ocimimum* spp. | 5CE1 | -10.1 | Rahman *et al*., 2020 |
| 16 | Hesperidin | *Citurs sp* | 1Z8G | -9.7 | Chikhale *et al*., 2020 |
| 17 | Qingdainone | *Strobilanthes cusia* | 1Z8G | -9.6 | Vivek-Ananth *et al*., 2020 |
| 18 | Edgeworoside C | *Edgeworthia gardneri* | 1Z8G | -9.6 | Vivek-Ananth *et al*., 2020 |
| 19 | Adlumidine | *Fumaria indica* | 1Z8G | -9.6 | Vivek-Ananth *et al*., 2020 |
| 20 | Pseudo-α-Colubrine | *Strychnos nux-vomica* | 1Z8G | -9.3 | Vivek-Ananth *et al*., 2020 |

**References**

Shawky, E., Ahmed A. Nadaa and Reham S. Ibrahim. Potential role of medicinal plants and their constituents in the mitigation of SARS-CoV-2: identifying related therapeutic targets using network pharmacology and molecular docking analyses. RSC Advances.2020;10,27961. doi.org/10.1039/D0RA05126H

Alamri MA, Altharawi A, Alabbas AB, Alossaimi MA, Alqahtani SM. Structure-based virtual screening and molecular dynamics of phytochemicals derived from Saudi medicinal plants to identify potential COVID-19 therapeutics. *Arabian Journal of Chemistry*. 2020;13(9):7224-7234. doi:10.1016/j.arabjc.2020.08.004

Vivek-Ananth, R. P., Rana, A., Rajan, N., Biswal, H. S., & Samal, A. (2020). In silico identification of potential natural product inhibitors of human proteases key to SARS-CoV-2 infection. Molecules,25, 3822.

Chikhale, R. V., Gupta, V. K., Eldesoky, G. E., Wabaidur, S. M., Patil, S. A., & Islam, M. A. (2020). Identification of potential anti-TMPRSS2 natural products through homology modelling, virtual screening and molecular dynamics simulation studies. Journal of Biomolecular Structure and Dynamics, 1-16.

Rahman, N., Basharat, Z., Yousuf, M., Castaldo, G., Rastrelli, L., & Khan, H. (2020). Virtual Screening of Natural Products against Type II Transmembrane Serine Protease (TMPRSS2), the Priming Agent of Coronavirus 2 (SARS-CoV-2). Molecules, 25(10), 2271.

Joshi, R. S. *et al*. Discovery of potential multi-target-directed ligands by targeting host-specific SARS-CoV-2 structurally conserved main protease. *J. Biomol. Struct. Dyn.* 1-16, https://doi.org/10.1080/07391102.2020.1760137 (2020).

Maurya, V. K., Kumar, S., Prasad, A. K., Bhatt, M. L. B. & Saxena, S. K. Structure-based drug designing for potential antiviral activity of selected natural products from Ayurveda against SARS-CoV-2 spike glycoprotein and its cellular receptor. *VirusDis.* 31, 179-193, https://doi.org/10.1007/s13337-020-00598-8 (2020).

Pandit, M. & Latha, N. *In silico* studies reveal potential antiviral activity of phytochemicals from medicinal plants for the treatment of COVID-19 infection. *Research Square* DOI: 10.21203/rs.3.rs-22687/v1 (2020).

Narkhede, Rohan R., Ashwini V. Pise, Rameshwar S. Cheke, and Sachin D. Shinde. "Recognition of natural products as potential inhibitors of COVID-19 main protease (Mpro): In-silico evidences." *Natural Products and Bioprospecting* (2020): 1-10. DOIhttps://doi.org/10.1007/s13659-020-00253-1

Gurung, Arun Bahadur *et al*. “Unravelling lead antiviral phytochemicals for the inhibition of SARS-CoV-2 M^pro^ enzyme through in silico approach.” *Life sciences* vol. 255 (2020): 117831. doi:10.1016/j.lfs.2020.117831

Majumder, Ranabir, and Mahitosh Mandal. "Screening of plant-based natural compounds as a potential COVID-19 main protease inhibitor: an in silico docking and molecular dynamics simulation approach." *Journal of Biomolecular Structure and Dynamics* (2020): 1-16. https://doi.org/10.1080/07391102.2020.1817787

Shivanika, C., Deepak Kumar, and Pawan Venkataraghavan Ragunathan. "Molecular docking, validation, dynamics simulations, and pharmacokinetic prediction of natural compounds against the SARS-CoV-2 main-protease." *Journal of biomolecular structure & dynamics*: 1-27. DOI: [10.1080/07391102.2020.1815584](https://doi.org/10.1080/07391102.2020.1815584)

Shree, Priya, *et al*. "Targeting COVID-19 (SARS-CoV-2) main protease through active phytochemicals of ayurvedic medicinal plants–Withania somnifera (Ashwagandha), Tinospora cordifolia (Giloy) and Ocimum sanctum (Tulsi)–a molecular docking study." *Journal of Biomolecular Structure and Dynamics* (2020): 1-14. DOI: 10.1080/07391102.2020.1810778.

Sharma, Priyanka, and Asifkhan Shanavas. "Natural derivatives with dual binding potential against SARS-CoV-2 main protease and human ACE2 possess low oral bioavailability: a brief computational analysis." *Journal of Biomolecular Structure and Dynamics* (2020): 1-12. doi: 10.1080/07391102.2020.1794970

Prasanth, D. S. N. B. K. et al. In silico identification of potential inhibitors from Cinnamon against main protease and spike glycoprotein of SARS CoV-2. J. Biomol. Struct. Dyn. 1-15, https://doi.org/10.1080/07391102.2020.1779129 (2020).

Tallei, T. E. et al. Potential of plant bioactive compounds as SARS-CoV-2 main protease (Mpro) and spike (S) glycoprotein inhibitors: A molecular docking study. Preprints 2020040102; https://doi.org/10.20944/preprints202004.0102.v2 (2020).

Ubani, A. et al. Molecular docking analysis of some phytochemicals on two SARS-CoV-2 targets: Potential lead compounds against two target sites of SARS-CoV-2 obtained from plants. bioRxiv 2020.03.31.017657; https://doi.org/10.1101/2020.03.31.017657 (2020).
